# Tau Exon 10 Inclusion by PrP^C^ through Downregulating GSK3β Activity

**DOI:** 10.3390/ijms22105370

**Published:** 2021-05-20

**Authors:** Laia Lidón, Laura Llaó-Hierro, Mario Nuvolone, Adriano Aguzzi, Jesús Ávila, Isidro Ferrer, José Antonio del Río, Rosalina Gavín

**Affiliations:** 1 Molecular and Cellular Neurobiotechnology, Institute for Bioengineering of Catalonia (IBEC), Scientific Park of Barcelona, The Barcelona Institute for Science and Technology (BIST), 08028 Barcelona, Spain; laia_211@hotmail.com (L.L.); laurallao143@gmail.com (L.L.-H.); 2 Department of Cell Biology, Physiology and Immunology, University of Barcelona, 08028 Barcelona, Spain; 3 Network Centre of Biomedical Research of Neurodegenerative Diseases (CIBERNED), Institute of Health Carlos III, Ministry of Economy and Competitiveness, 28031 Madrid, Spain; javila@cbm.csic.es (J.Á.); 8082ifa@gmail.com (I.F.); 4 Institute of Neuroscience, University of Barcelona, 08035 Barcelona, Spain; 5 Amyloidosis Research and Treatment Center, Foundation IRCCS Policlinico San Matteo, Department of Molecular Medicine, University of Pavia, 27100 Pavia, Italy; mario.nuvolone@usz.ch; 6 Institute of Neuropathology, University Hospital of Zurich, 8091 Zurich, Switzerland; adriano.aguzzi@usz.ch; 7 Center for Molecular Biology “Severo Ochoa” (CSIC-UAM), 28049 Madrid, Spain; 8 Department of Pathology and Experimental Therapeutics, University of Barcelona, 08907 Barcelona, Spain; 9 Bellvitge Biomedical Research Centre, Bellvitge University Hospital, IDIBELL, 08908 Barcelona, Spain

**Keywords:** cellular prion protein, GSK3β, microtubule-associated protein tau, alternative splicing, Alzheimer’s disease, tauopathies

## Abstract

Tau protein is largely responsible for tauopathies, including Alzheimer’s disease (AD), where it accumulates in the brain as insoluble aggregates. Tau mRNA is regulated by alternative splicing, and inclusion or exclusion of exon 10 gives rise to the 3R and 4R isoforms respectively, whose balance is physiologically regulated. In this sense, one of the several factors that regulate alternative splicing of tau is GSK3β, whose activity is inhibited by the cellular prion protein (PrP^C^), which has different physiological functions in neuroprotection and neuronal differentiation. Moreover, a relationship between PrP^C^ and tau expression levels has been reported during AD evolution. For this reason, in this study we aimed to analyze the role of PrP^C^ and the implication of GSK3β in the regulation of tau exon 10 alternative splicing. We used AD human samples and mouse models of PrP^C^ ablation and tau overexpression. In addition, we used primary neuronal cultures to develop functional studies. Our results revealed a paralleled association between PrP^C^ expression and tau 4R isoforms in all models analyzed. In this sense, reduction or ablation of PrP^C^ levels induces an increase in tau 3R/4R balance. More relevantly, our data points to GSK3β activity downstream from PrP^C^ in this phenomenon. Our results indicate that PrP^C^ plays a role in tau exon 10 inclusion through the inhibitory capacity of GSK3β.

## 1. Introduction

Tauopathies are a group of neurodegenerative diseases characterized by the presence of intracellular aggregates of hyperphosphorylated tau [1,2]. Among others, the group includes: frontotemporal dementia and parkinsonism linked to chromosome 17 (FTDP-17), Pick’s disease, corticobasal degeneration (CBD), progressive supranuclear palsy (PSP), and Alzheimer’s disease (AD) [2,3,4,5]. AD is also characterized by the presence of senile plaques, composed mainly of extracellular deposits of β-amyloid (Aβ) peptide generated by the sequential proteolysis of the amyloid precursor protein (APP) by β- and γ-secretases [6]. The amyloid hypothesis suggests that Aβ deposition in brain parenchyma triggers a sequence of events leading to tau dysfunction [7,8]. Meanwhile, accumulation of hyperphosphorylated tau is decisive for the progression of AD, following the defined Braak stages [9].

Tau is a neuronal microtubule-associated protein, encoded by the *MAPT* gene in humans, which promotes the polymerization and stabilization of microtubules (MT) under the regulatory control of several kinases and phosphatases. In fact, phosphorylation of tau inhibits its binding to MT in a physiological and controlled way [10], but pathological hyperphosphorylation generates tau aggregates into paired helical filaments (PHF) and later into neurofibrillary tangles (NFTs). This leads to increased MT instability, impaired axonal transport, and profound deficits in synaptic function [11]. The *MAPT* gene is transcribed under a complex alternative splicing of exons 2, 3, and 10, generating 6 possible isoforms. Three of them, named 4R tau for inclusion of exon 10, confer a great level of MT binding to the tau protein [12], while the absence of exon 10 generates the three isoforms named 3R tau, more susceptible to phosphorylation [10]. In addition, 4R tau has been implicated in neuronal maturation [13], while 3R tau is predominant during embryonic development and specific neuronal types [14,15]. Equal levels of 3R and 4R tau are expressed in the adult human brain [16], but the 3R/4R tau balance is altered in brains affected by several tauopathies, showing the importance of dysregulation of tau exon 10 alternative splicing in neurodegeneration [17,18]. In addition, several factors are involved in the complex control of exon 10 splicing and tau metabolism (see [19] for review). Among others, the glycogen synthase kinase 3-β (GSK3β), which also phosphorylates tau in healthy brains [20], represents one of these factors [21].

The cellular prion protein (PrP^C^), highly expressed by neurons and glial cells in the adult central nervous system (CNS) [22,23,24], has been extensively studied as the causal agent of transmissible spongiform encephalopathies (TSEs) when it is abnormally processed as the proteinase-K resistant PrP^Sc^ isoform [25]. However, instead, its physiological function in the brain seems to have a neuroprotective role [26,27,28]. In fact, one of the best-defined functions of PrP^C^ is its antioxidant activity though different means, including copper homeostasis [29], modulation of endogenous superoxide dismutase (SOD) [30], and glutathione reductase (GR) activities [31]. Nevertheless, an increasing number of studies suggest that PrP^C^ is involved in neuronal differentiation of neural progenitors from different stem cells populations [32,33,34], a process strongly influenced by GSK3 activity [35]. In this sense, PrP^C^ triggers in vivo reduction of GSK3β kinase activity through phosphorylation of GSK3β on serine 9 residue [36].

Despite the putative participation of PrP^C^ in β-amyloid mediated pathology in AD [37,38,39,40,41], we reported the neuroprotective role of PrP^C^ in the modulation of tau levels in various models of the disease. In this sense, we illustrated a greater susceptibility of *Prnp*^0/0^ primary cultures to tau overexpression and phosphorylation under treatment of Aβ-derived diffusible ligands (ADDLs), the parallel progression of PrP^C^ and tau expression in APP/PS1 mice, and the inverse correlation between levels of PrP^C^ and tau in postmortem AD brains (ranking from Braak stage I to stage VI) [42]. In addition, the study ruled out participation of PrP^C^ in the promoter activity of tau, suggesting additional mechanisms of control, probably at post-transcriptional level, that may be analyzed. Indeed, an increase in 3R/4R tau ratio is observed in the analysis of induced pluripotent stem cells (iPSCs) derived from a Gertsmann–Straussler–Scheinker syndrome (GSS) patient carrying the *Y218N PRNP* mutation. In addition, we recently reported the modulation of *PRNP* promoter activity by tau [41], supporting the physiological contribution of PrP^C^ in tau biology. Thus, in this study we investigate the putative participation of PrP^C^ in the alternative splicing of tau exon 10, both at the physiological level and in the disease, using mouse models and AD brains. Our results indicate that PrP^C^ plays a role in tau exon 10 inclusion through GSK3β inhibitory capacity.

## 2. Results

### 2.1. Increased 3R/4R Tau Ratio in Mice Lacking PrP^C^

**Figure 1:**
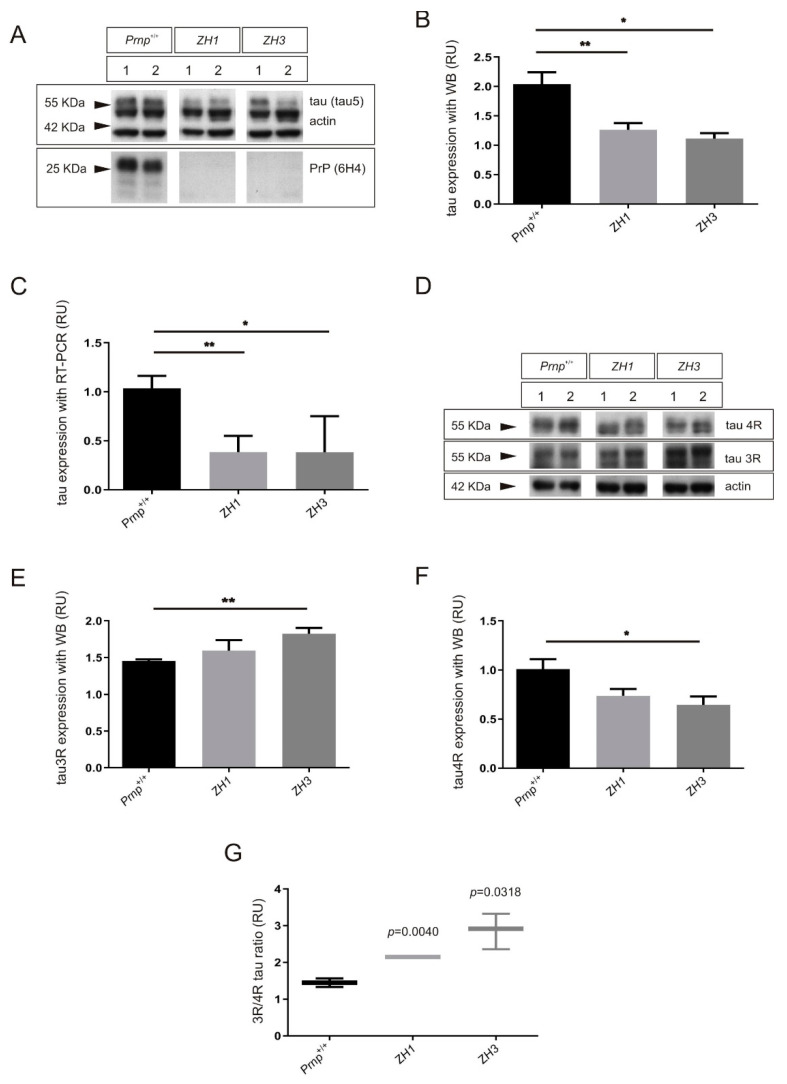
Effects on tau and its alternative exon 10 isoforms in mice devoid of PrP^C^ expression. (**A**–**C**) Total tau expression analyzed in brain extract from WT and *Prnp*^0/0^ mice ZH1 or ZH3 at the age of 3 months. (**A**) Representative WB analysis using total anti-tau antibody (monoclonal Tau5) in parallel with anti-PrP^C^ antibody (monoclonal 6H4) in each case. Actin detection was used as control loading protein. (**B**) Histograms showing the densitometry study of tau expression in each genotype. (**C**) Histograms showing the RT-qPCR analysis of expression of tau in mice analyzed in (**A**). (**D**–**G**) 3R and 4R tau isoform expression analyzed in brain extract from WT and *Prnp*^0/0^ mice ZH1 or ZH3 at the age of 3 months. (**D**) Representative WB analysis using anti-3R tau antibody (monoclonal RD3) in parallel with anti-4R tau antibody (monoclonal RD4) in each genotype. Actin detection was used as control loading protein. (**E**,**F**) Histograms showing the densitometry study of 3R tau (**E**) or 4R tau (**F**) expression in each genotype. (**G**) Graphical representation of the 3R/4R tau ratio analyzed with data represented in (**E**,**F**). Between 3 and 5 mice were examined in each group and data represents the mean ± S.E.M. Differences between groups were considered statistically significant at ** p < 0.01 and * p < 0.05 (*t*-test).

Taking into account that PrP^C^ plays an inhibitory role in GSK3β activity [36], it may have an impact on tau exon 10 alternative splicing [43]. To further explore the physiological role of PrP^C^ in inclusion or exclusion of tau exon 10, we analyzed tau expression, both total amount of protein and relative spliced isoforms, in brain samples of mice lacking PrP^C^ (Figure 1). Thus, we used two PrP^C^-null mouse strains; ZH1, which presents a mixed genetic background, and ZH3, a co-isogenic mouse (see Material and Methods for more information). Between three and five mice were analyzed in each case at the age of 3 months. Results obtained with western blot (WB) analysis showed a 1.64-fold decrease (** *p* = 0.003) in total tau levels of ZH1 mice and a significant 1.83-fold decrease (* *p* = 0.011) in ZH3 when compared with *Prnp*^+/+^ mice (Figure 1A,B). These correlated with RT-PCR results that showed a significant fold decrease of mRNA tau levels, both in ZH1 (2.69, ** *p* = 0.001) and ZH3 (2.71, * *p* = 0.046) when compared with wild type (WT) (Figure 1C). 

Next, we analyzed the 3R and 4R tau isoforms in each mouse model with WB analysis (Figure 1D–G). Developed films showed a tendency to increase in 3R tau expression of ZH1 mice (1.10, *p* = 0.281) and a significant 1.26-fold increase (** *p* = 0.0087) in ZH3 when compared with *Prnp*^+/+^ mice (Figure 1D,E). In addition, the analysis of 4R tau expression showed a tendency to decrease in ZH1 mice (1.38, *p* = 0.056) and a significant 1.56-fold decrease (* *p* = 0.033) in ZH3 when compared with WT (Figure 1D,F). Consequently, the 3R/4R tau ratio was significantly increased in mice lacking PrP^C^, both in ZH1, with a 1.49-fold increase (** *p* = 0.004) and in ZH3, with a 1.98-fold increase (* *p* = 0.0318) (Figure 1G). 

**Figure 2:**
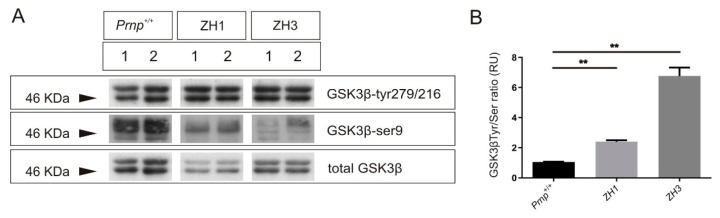
GSK3β activity in mice devoid of PrP^C^ expression. (**A**,**B**) GSK3β activation analyzed by WB in brain extract from WT and *Prnp*^0/0^ mice ZH1 or ZH3 at the age of 3 months. (**A**) Representative WB analysis using anti-phospho-tyr^279/216^ GSK3 antibody (monoclonal 5G-2F) in parallel with anti-phospho-ser^9^ GSK3 antibody (monoclonal 2D3) in each case. Membranes were re-probed with antibody against total GSK3 (monoclonal 4G-1E) for protein standardization. (**B**) Histograms showing the quantified ratio between phospho-tyr^279/216^ and phospho-ser^9^ after densitometry analysis of both phosphorylated GSK3β epitopes in each genotype, which represents the kinase activity. *n* = 3 mice were examined in each group and data represent the mean ± S.E.M. Differences between groups were considered statistically significant at ** p < 0.01 (*t*-test).

Finally, we aimed to analyze GSK3β activity after PrP^C^ ablation in our animal models. Densitometric results of WB analysis of GSK3β phosphorylation at tyr^279/216^ and ser^9^ showed a significant fold increase in the tyr^279/216^/ser^9^ ratio in both PrP^C^ knock-out mouse models when compared to *Prnp*^+/+^ mice (Figure 2) (2.28, ** *p* = 0.0021 for ZH1 and 6.42, ** *p* = 0.0044 for ZH3). This indicates raised GSK3β activity after PrP^C^ ablation. 

### 2.2. PrP^C^ Ablation Modifies the 3R/4R Tau Ratio in Mouse Models of Tau Overexpression

**Figure 3:**
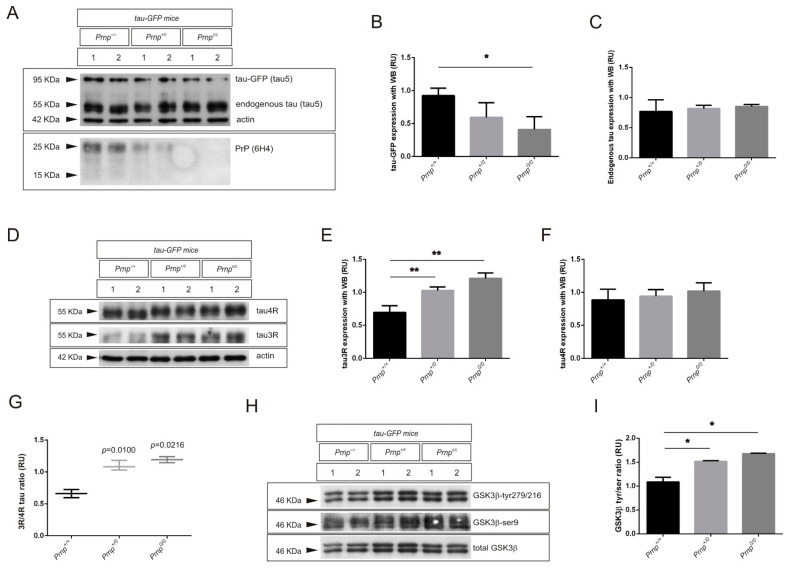
Effects on tau, alternative exon 10 forms, and GSK3β in tau transgenic mice overexpressing tau-GFP after ablation of PrP^C^ expression. (**A**–**C**) Total tau expression analyzed in brain extract from tau-GFP, tau-GFP-*Prnp*^+/0^, and tau-GFP-*Prnp*^0/0^ mice at the age of 3 months. (**A**) Representative WB analysis using total anti-tau antibody (monoclonal Tau5) in parallel with anti-PrP^C^ antibody (monoclonal 6H4) in each case. Actin detection was used as control loading protein. (**B**) Histograms showing the densitometry study of tau-GFP expression in each genotype. (**C**) Histograms showing the densitometry study of endogenous tau expression in each genotype. (**D**–**G**) Endogenous 3R and 4R tau isoforms expression analyzed in brain extract from tau-GFP, tau-GFP-*Prnp*^+/0^, and tau-GFP-*Prnp*^0/0^ mice at the age of 3 months. (**D**) Representative WB analysis using anti-3R tau antibody (monoclonal RD3) in parallel with anti-4R tau antibody (monoclonal RD4) in each genotype. Actin detection was used as control loading protein. (**E**,**F**) Histograms showing the densitometry study of 3R tau (**E**) and 4R tau (**F**) expression in each genotype. (**G**) Graphical representation of the 3R/4R tau ratio analyzed with data represented in (**E**,**F**). (**H**,**I**) GSK3β activation analyzed with WB in brain extract from tau-GFP, tau-GFP- *Prnp*^+/0^, and tau-GFP-*Prnp*^0/0^ mice at the age of 3 months. (**H**) Representative WB analysis using anti-phospho-tyr^279/216^ GSK3 antibody (monoclonal 5G-2F) in parallel with anti-phospho-ser^9^ GSK3 antibody (monoclonal 2D3) in each case. Membranes were re-probed with antibody against total GSK3 (monoclonal 4G-1E) for protein standardization. (**I**) Histograms showing the quantified ratio between phospho-tyr^279/216^ and phospho-ser^9^ after densitometry analysis of both phosphorylated GSK3β epitopes in each genotype, which represents the kinase activity. *n* = 3 mice were examined in each group and data represents the mean ± S.E.M. Differences between groups were considered statistically significant at ** p < 0.01 and * p < 0.05 (*t*-test).

**Figure 4:**
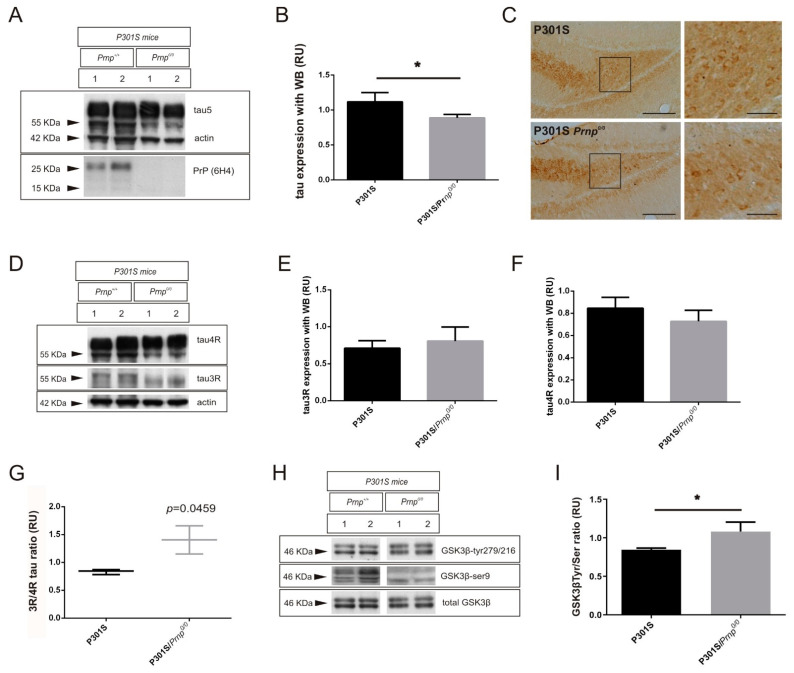
Effects on tau, alternative exon 10 forms, and GSK3β in tau transgenic mice overexpressing human P301S *MAPT* mutation after ablation of PrP^C^ expression. (**A**–**C**) Total tau expression analyzed in brain extract from P301S and P301S-*Prnp*^0/0^ mice at the age of 3 months. (**A**) Representative WB analysis using total anti-tau antibody (monoclonal Tau5) in parallel with anti-PrP^C^ antibody (monoclonal 6H4) in each case. Actin detection was used as control loading protein. (**B**) Histograms showing the densitometry study of both endogenous and overexpressed tau expression in each genotype. (**C**) Immunohistochemical detection of total tau in mouse brain sections from P301S and P301S-*Prnp*^0/0^. Monoclonal tau5 antibody was used to detect increased immunoreaction in DG from P301S in contrast to P301S-*Prnp*^0/0^ animals. Scale bars = 100 µm and 50 µm. (**D**–**G**) Endogenous 3R and 4R tau isoform expression analyzed in brain extract from P301S and P301S-*Prnp*^0/0^ mice at the age of 3 months. (**D**) Representative WB analysis using anti-3R tau antibody (monoclonal RD3) in parallel with anti-4R tau antibody (monoclonal RD4) in each genotype. Actin detection was used as control loading protein. (**E**,**F**) Histograms showing the densitometry study of 3R tau (**E**) and 4R tau (**F**) expression in each genotype. (**G**) Graphical representation of the 3R/4R tau ratio analyzed with data represented in (**E**,**F**). (**H**,**I**) GSK3β activation analyzed with WB in brain extract from P301S and P301S-*Prnp*^0/0^ mice at the age of 3 months. (**H**) Representative WB analysis using anti-phospho-tyr^279/216^ GSK3 antibody (monoclonal 5G-2F) in parallel with anti-phospho-ser^9^ GSK3 antibody (monoclonal 2D3) in each case. Membranes were re-probed with antibody against total GSK3 (monoclonal 4G-1E) for protein standardization. (**I**) Histograms showing the quantified ratio between phospho-tyr^279/216^ and phospho-ser^9^ after densitometry analysis of both phosphorylated GSK3β epitopes in each genotype, which represents the kinase activity. *n* = 3 mice were examined in each group and data represents the mean ± S.E.M. Differences between groups were considered statistically significant at * p < 0.05 (*t*-test).

Next, we analyzed the effects of the loss of function of PrP^C^ on overexpressed tau in transgenic mouse models. Thus, TgTP6.3 and P301S mouse lines were backcrossed respectively with *Prnp*^0/0^ mice. In this line, we decided to work with co-isogenic ZH3, which presents significant changes in spliced isoforms and higher altered 3R/4R tau ratio than ZH1 as observed before, and to avoid the mixed background of the ZH1 mice. Then, PrP^C^ knock-out animals with overexpression of non-mutated tau (Figure 3) or human P301S mutated tau (Figure 4) were sacrificed at the age of 3 months and analyzed for total tau levels, 3R and 4R tau splicing isoforms, and GSK3β activity, with WB (*n* = 3/group).

After densitometric analysis of developed films, tau-GFP overexpressed from tau-GFP mice (TgTP6.3) showed a progressive reduction with the lack of one or two copies of the *Prnp* gene (Figure 3A,B). In fact, tau-GFPx*Prnp*^0/0^ showed a significant 2.25-fold decrease (* *p* = 0.0308) in tau-GFP when compared to tau-GFPx*Prnp*^+/+^ (Figure 3B). In addition, no significant changes in endogenous tau expression, both in heterozygous and non-homozygous *Prnp* mice were found (Figure 3A,C). However, PrP^C^ levels were reverse correlated with endogenous 3R tau levels (Figure 3D,E), which resulted in a significant fold-increase of 1.48 (** *p* = 0.0073) or 1.75 (** *p* = 0.0099) in tau-GFP heterozygous or homozygous for *Prnp* respectively. As a consequence, and even though endogenous 4R tau levels remained unchanged (Figure 3F), the 3R/4R tau ratio was significantly altered by loss of PrP^C^, with a 1.66-fold increase in tau-GFPx*Prnp*^+/0^ mice (* *p* = 0.010) and a 1.80-fold increase in tau-GFPx*Prnp*^0/0^ mice (* *p* = 0.0216) (Figure 3G). As expected, the activation of GSK3β by WB was significantly increased with the lack of one or two copies of the gene (Figure 3H,I). In this sense, tau-GFPx*Prnp*^+/0^ and tau-GFPx*Prnp*^0/0^ mice presented a 1.40-fold increase (* *p* = 0.0490) and 1.55-fold increase (* *p* = 0.0268) respectively (Figure 3I).

In contrast, when we analyzed the lack of PrP^C^ function on a tau mutated form (human P301S), we also observed a significant 1.26-fold decrease (* *p* = 0.0472) in total tau levels with WB in mice lacking PrP^C^ expression, and in this case both endogenous (lower band of around 55 KDa) and overexpressed (upper band of 70 KDa) (Figure 4A,B). In addition, immunohistochemical analysis of P301Sx*Prnp*^0/0^ animals showed a slight decrease in Tau5 staining in the dentate gyrus (Figure 4C). Lastly, and taking into account that the mutant tau band is a 4R form, we analyzed endogenous tau splicing isoforms (both 3R and 4R) with WB (Figure 4D–G). Although results for individual isoforms were not significantly changed in PrP^C^ knock-out mice (Figure 4D–F), the 3R/4R tau ratio from P301Sx*Prnp*^0/0^ mice presented a significant 1.69-fold increase (* *p* = 0.0459) when compared to P301S (Figure 4G). In addition, WB analysis of the GSK3β tyr/ser ratio suggested a correlation between the 3R/4R tau ratio and kinase activation, as P301Sx*Prnp*^0/0^ mice showed a significant 1.28-fold increase (* *p* = 0.0319) when compared to P301Sx*Prnp*^+/+^ (Figure 4H,I). 

Taken together, our results indicated that in a similar manner to ZH1 and ZH3 animals, loss of PrP^C^ induced a significant reduction of total tau expression both in a mutated and non-mutated tau transcript, and a significant alteration of endogenous 3R/4R tau ratio in favor of 3R isoforms in both models.

### 2.3. Tau Exon 10 Splicing Is Dependent on PrP^C^ Dosage in Correlation with GSK3β

To further explore the participation of PrP^C^ in tau exon 10 splicing through GSK3β activity, we developed in vitro functional experiments. By analogy, we investigated an experimental model of cortical primary cultures, because both PrP^C^ and 4R tau are directly implicated in neuronal differentiation. 

**Figure 5:**
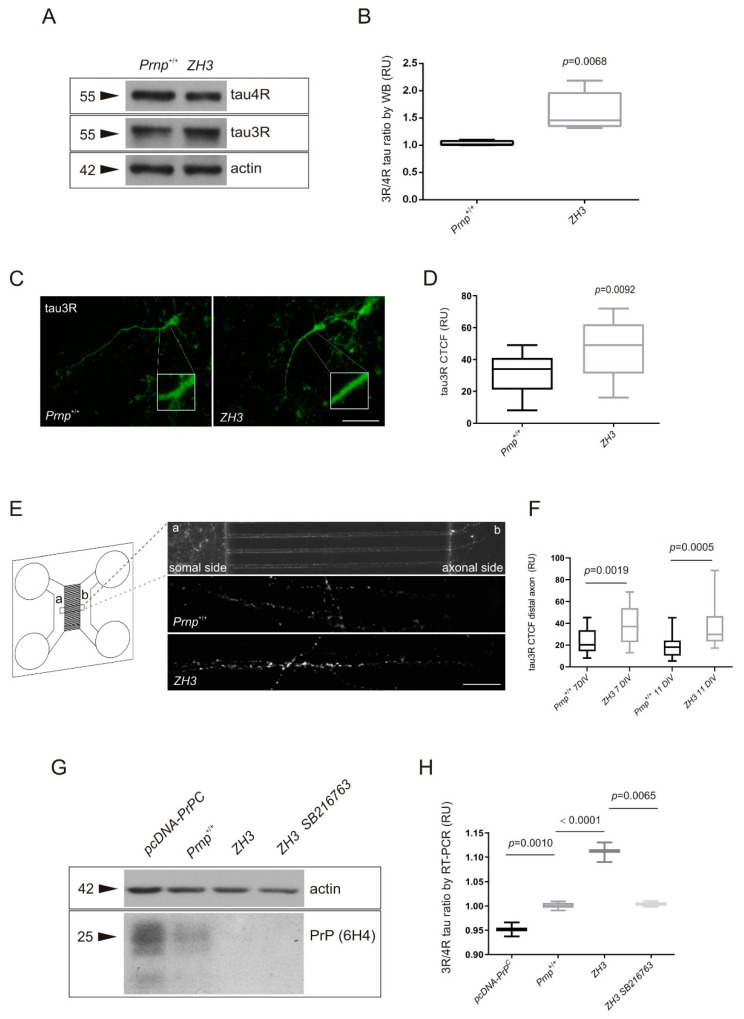
Effects on alternative exon 10 tau splicing downstream GSK3β activity in cortical cultures from WT and ZH3 mice depending on GSK3β activity condition. (**A**–**F**) 3R and 4R tau isoform expression analyzed in cortical primary cultures from WT (*Prnp*^+/+^) and *Prnp*^0/0^ (ZH3) mice. (**A**) Representative WB analysis using anti-3R tau antibody (monoclonal RD3) in parallel with anti-4R tau antibody (monoclonal RD4) in primary cultures from each genotype at 7 *DIV*. Actin detection was used as control loading protein. (**B**) Graphical representation of the 3R/4R tau ratio analyzed in *n* = 3 independent primary cultures from each genotype. (**C**) Representative immunocytochemical detection of 3R tau isoforms in neurites of cultured neurons from *Prnp*^+/+^ and ZH3 mice at 7 *DIV*. Monoclonal RD3 antibody was used to detect an increased immunoreaction in ZH3 in contrast to *Prnp*^+/+^ animals. The square represents the proximal neurite region used to quantify fluorescence. Scale bar = 50 µm. (**D**) CTCF values derived from immunofluorescence microphotographs of 3R tau expression in neurites derived from cultured neurons of *Prnp*^+/+^ and ZH3 mice. The mean ± S.E.M. from each genotype was obtained after quantifying three different points inside the square of the image from *n* = 4 neurons for each culture. (**E**) Representation of the PDMS devices used to isolate axons of cultured neurons from *Prnp*^+/+^ and ZH3 mice at 7 and 11 *DIV* (see the somal side (a reservoir) and axonal side (b reservoir) and magnified examples of 3R tau-labelled at the axonal side at 7 *DIV*). Scale bar = 25 µm. (**F**) CTCF values derived from immunofluorescence microphotographs of 3R tau expression in distal axons shown in (**E**) and derived from cultured neurons of *Prnp*^+/+^ and ZH3 mice. The mean ± S.E.M. from each genotype and *DIV* was obtained after quantifying one to five individual axons from ten microphotographs. (**G**,**H**) Changes in 3R and 4R tau isoform expression analyzed in cortical primary cultures from WT (*Prnp*^+/+^) and *Prnp*^0/0^ (ZH3) mice at 7 *DIV*. (**G**) Representative WB analysis using anti-PrP^C^ antibody (monoclonal 6H4) in primary cultures after modifying GSK3β activity by overexpressing PrP^C^ or use of the GSK3β inhibitor SB-216763. Actin detection was used as a control loading protein. (**H**) Graphical representation of the 3R/4R tau ratio analyzed from *n* = 3 independent primary cultures from each GSK3β activity condition. *p* values indicating statistical differences between groups were determined using *t* test.

A total of three independent primary cortical cultures from ZH3 mice were analyzed after 7 days in vitro (*DIV*). Results obtained with WB showed a significant fold increase of the 3R/4R tau ratio in ZH3 (1.56, ** *p* = 0.0068) when compared with WT (Figure 5A,B) in correlation with changes in brain homogenates from mice of the same genotype. Next, in an attempt to analyze individual neurons, corrected total cell fluorescence measurement (CTCF, see [44]) was taken on individual primary neurons at 11 *DIV* to quantify 3R tau intensity after the immunocytochemical procedure. Neurons blind to the genotype of the mouse were selected from three independent primary cultures. Then, CTCF was measured both in soma and in a proximal neurite region (three successive counts along the area inside the box (Figure 5C)). Although non-significant differences were found between ZH3 and *Prnp*^+/+^ mice for soma CTCF, 3R tau signal intensity showed a 1.49-fold increase (** *p* = 0.0092) in ZH3 neurites when compared to *Prnp*^+/+^ (Figure 5C,D). In addition, differences in 3R tau expression of both genotypes was confirmed on isolated cultured axons at 7 and 11 *DIV*. To do this, microfluidic PDMS devices were used (see Material and Methods for more information). Then, cortical cultures were added to the somal-side reservoir (a) and neurons projected axons into the axonal side (b) though central channels (Figure 5E). One to five isolated axons blind to the genotype of the mouse were selected from ten images of each genotype to measure CTCF, determining the same area of measurement in each case. As a result, 3R tau signal intensity showed a 1.63-fold increase (** *p* = 0.0019) in ZH3 axons when compared to *Prnp*^+/+^ at 7 *DIV* and a 2.07-fold increase (*** *p* = 0.0005) in ZH3 axons when compared to *Prnp*^+/+^ at 11 *DIV* (Figure 5F).

Finally, we developed functional experiments in two ways; first, to investigate the effect of modifying PrP^C^ dosage on the 3R/4R tau ratio, and second to correlate this effect with GSK3β activity. Then, overexpression of PrP^C^ was induced in primary cortical cultures by transfection with pcDNA-PrP^C^ while SB216763 was used as inhibitor of GSK3β (Figure 5G). After that, RT-PCR analysis was performed to analyze the 3R and 4R balance for each experimental condition (Figure 5H). Results of three independent experiments showed a significant increase in the 3R/4R tau ratio in ZH3 cultures (1.11, **** *p* < 0.0001) when compared to WT cultures (Figure 5H) as previously described when we analyzed tau isoforms with WB (Figure 5B). Otherwise, a slight overexpression of PrP^C^ (of around 15 percent, see Figure 5G) induced a 1.05-fold decrease (** *p* = 0.001) of the 3R/4R tau ratio when compared to WT cultures (Figure 5H). When analyzing tau splicing in ZH3 cortical cultures treated with SB216763, which inhibited GSK3β activity around 40 percent (Figure S1), we found a 1.11-fold decrease (** *p* = 0.0065) in the 3R/4R tau ratio when compared to ZH3 cultures (Figure 5H). Therefore, ZH3 cultures treated with SB216763 presented similar splicing levels to *Prnp*^+/+^ cultures, suggesting PrP^C^ regulation of tau splicing through GSK3β.

### 2.4. Impact of PrP^C^ Levels on Tau Splicing in AD Brain 

**Figure 6:**
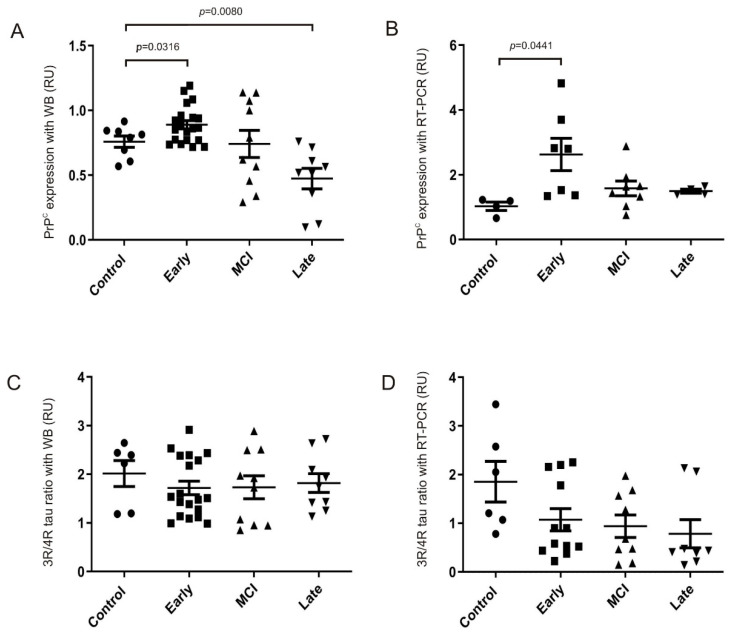
Expression of PrP^C^ and splicing exon 10 isoforms of tau in AD brains according to Braak stage. (**A**,**B**) Plots illustrating PrP^C^ expression by WB using anti-PrP^C^ antibody (monoclonal 6H4). Actin detection was used as a control loading protein (**A**) or RT-PCR (**B**) in cases shown in Table 1 and grouped as Non-AD, Initial (Braak I-II), Intermediate (Braak III-IV), or Late AD (Braak V-VI). (**C**,**D**) Plots illustrating the ratio between 3R and 4R tau isoforms with WB analysis with RD3 and RD4 antibodies and using actin as control loading protein (**C**) or RT-PCR (**D**) in cases shown in Table 1. Each dot corresponds to one sample and the mean ± S.E.M. for each group is also displayed.

Many efforts have been made to understand pathological causes and consequences of altered 3R/4R tau ratios in tauopathies. Thus, analyses of tau mis-splicing have been reported in AD-affected brains showing no alteration of the 3R/4R tau ratio in disease [3,45,46]. However, these studies were focused on advanced AD Braak stages (from III to VI). In addition, the potential of PrP^C^ to control some factors involved in splicing of exon 10 in disease has not been analyzed to date. Initially we aimed to determine whether overexpression of PrP^C^ at initial AD Braak stages (from I to II) might affect not only tau levels [42,47] but also tau splicing profile. Thus, samples referred to in Table 1 were analyzed for expression levels of 3R and 4R tau isoforms at different stages to correlate with PrP^C^ expression levels (Figure 6A–D). As indicated in the table, some samples were analyzed with WB, RT-PCR, or both techniques depending on availability of tissue. Then, in order to analyze results, the Braak stages were grouped into Initial (Braak I-II), Intermediate (Braak III-IV), and Late (Braak V-VI) where Initial corresponds to stages with greater PrP^C^ expression levels in contrast to Late, which shows decreased PrP^C^ expression (Figure 6A,B). Results showed a tendency to decrease the 3R/4R tau ratio 1.17-fold (*p* = 0.3194), with WB (Figure 6C), or decrease 1.73-fold (*p* = 0.0912) with RT-PCR (Figure 6D), at Initial AD stages, which reverse correlates with PrP^C^, which showed a 1.17-fold increase (* *p* = 0.0316) with WB (Figure 6A), or a 2.55-fold increase (* *p* = 0.0441) with RT-PCR (Figure 6B), when compared to non-degenerative (non-AD) controls. However, the 3R/4R tau ratio remained unchanged or decreased (by WB or RT-PCR respectively) during AD progression (Figure 6C,D), even though the high levels of PrP^C^ are no longer maintained (Figure 6A,B). Interestingly, the ratio of GSK3β-pTyr/pSer analyzed with WB progresses, although in a non-significant manner, in reverse correlation with PrP^C^ levels analyzed with WB in the same samples (Figure 6A), as expected (Figure S2). Therefore, upregulation of PrP^C^ levels on Initial stage samples (Figure 6A) is reverse correlated with a 1.31-fold decrease (*p* = 0.1409) in GSK3β activity (Figure S2), while there is a tendency to recover GSK3β activity levels as PrP^C^ levels decrease in AD progression (Figure 6A and Figure S2). Thus, taking into account the extensive list of factors that regulate the splicing of exon 10 of *MAPT* pre-mRNA [19], we selected and analyzed the expression of miR132-3p because some authors have reported down-regulation of this microRNA in AD progression [48,49]. Both human samples and the three animal models used in this study were analyzed with RT-PCR and, as shown in Figure S3, only hippocampal human samples showed, although without statistical significance, a tendency to down-regulation in a progressive manner from non-AD to Late AD affected brains, and independently of PrP^C^ levels (Figure S3A). In this line, the fold-decrease between non-AD and other groups was 1.27 (*p* = 0.3152) to Initial, 1.43 (*p* = 0.1347) to Intermediate, and 1.62 (*p* = 0.1995) to Late, respectively. In addition, none of the animal models showed alterations of miR132-3p levels in a PrP^C^-dependent manner (Figure S3B–D). Altogether, these results suggest that although GSK3 activity is affected in AD progression by changing levels of PrP^C^, this relation is not mandatory in dysregulation of tau alternative splicing in the disease. 

## 3. Discussion

Tau exon 10 alternative splicing is physiologically regulated during human brain development and in neuronal differentiation [50,51]. While 0N3R tau is expressed only in fetal stages, the CNS expresses all six isoforms, and 3R and 4R tau are present in equal amounts under normal conditions [50]. In the adult brain, pathological dysregulation that results in imbalance of 3R and 4R tau expression contributes to neurofibrillary degeneration, a hallmark of AD and other tauopathies [19,52,53]. Importantly, PrP^C^ expression is also regulated during development [54] and promotes neuronal differentiation [55,56]. In addition, PrP^C^ inihibits GSK3β activity [36], and the kinase is proposed as playing a central role in AD under the GSK3 hypothesis [57] as it is involved in the mechanisms underlying learning and memory, the hyperphosphorylation of tau, the increased production of Aβ, local cerebral inflammatory responses, and finally in tau exon 10 splicing [19,21,43]. In fact, the neuroprotective role of GSK3 inhibitors in cellular and animal models of AD has been widely reported [58,59,60,61] and it continues to be a topic of study and clinical investigation [62]. To date, the consequences of the PrP^C^ inhibitory effect on GSK3β have been associated with PrP^C^-STI-1 interaction, which leads to neuronal protection [36], through the (i) stress protective role of both PrP^C^ and STI-1 [26,27], (ii) the pivotal role of GSK3β in synaptic plasticity and long-term potentiation (LTP) [63], and (iii) the countering of Aβ oligomer toxicity [64]. However, GSK3β inhibition with PrP^C^ had never been related to tau splicing before. Thus, as PrP^C^ effects on tau are not fully determined [42], in the present study we investigated the possibility that PrP^C^ is involved in splicing of tau exon 10 through GSK3β. 

### 3.1. Increase in the 3R/4R Tau Ratio Paralleled to GSK3β Activation in Mouse Models

In the adult mouse brain, tau exon 10 becomes constitutive [65]. However, McMillan et al. reported two splicing forms in region- and cell-specific manners [66], confirming 3R tau isoform detection in adult mice. We were able to detect both tau isoforms in adult brain extract from all of the mouse models used in this study with WB. Furthermore, both the 3R and 4R tau isoforms have also been detected in primary cultures with WB and RT-PCR. Thus, our results revealed that lack of PrP^C^ expression in adult mouse brain resulted in a significantly increased ratio of 3R/4R tau isoforms in all mouse models analyzed despite different expression of independent isoforms in each model. In this sense, we found that 3R tau expression was slightly increased in the ZH1 model while significantly overexpressed in ZH3. In addition, both strains presented decreased 4R tau expression, but this was only significant for ZH3. In fact, *Prnp*-linked loci polymorphisms as a consequence of ZH1 generation constitute systematic experimental confounders [67], which could explain the differences between the two models. Regarding tau-GFP mice, we found that lack of PrP^C^ expression induced an increase in endogenous 3R tau isoforms but not changes in endogenous 4R tau isoforms, while levels of tau-GFP expression, exclusively 4R, were significantly decreased. Lastly, changes in individual tau isoforms from P301S/*Prnp*^0/0^ showed only a tendency to increase, in the case of 3R tau, and to decrease, in the case of 4R tau when compared to P301S. However, the resulting quantitative 3R/4R tau ratio was also significantly increased. Taken together, our results point to a misbalance in spliced tau isoforms in favor of 3R tau levels promoted by lack of PrP^C^ expression. Importantly, and taking into account that overexpressed forms in both transgenic models are exclusively 4R, the fact that in both cases they are diminished by the lack of PrP^C^ expression supports the role of PrP^C^ in post-transcriptional mechanisms of tau regulation such as alternative splicing. On the other hand, it is well known that changes in tau ratio isoforms modify tau-MT affinity, presenting the 3R tau isoforms with a decreased ability to interact with MT [12,68,69], and affecting as a consequence neuronal events such as the axonal transport capability between others [70,71]. In this sense, we and others recently reported decreased α-synuclein spreading in *Prnp*^0/0^ mice as a consequence of reduced binding of α-synuclein to the plasma membrane [72,73], although the present results suggest that a misbalance in tau isoforms in mice lacking PrP^C^ may also be involved in axon transport capability. 

Despite this, our knowledge of the *Prnp*^0/0^ phenotype shows that adult *Prnp*^0/0^ mice may have compensatory mechanisms minimizing the lack of PrP^C^ expression, which are also strain-dependent [74,75]. Therefore, we transferred our findings to cortical primary cultures, where active processes of neuronal maturation occur, and we also found an increase in 3R isoform expression and a consequent increased 3R/4R tau ratio in the absence of PrP^C^ expression in cultured ZH3 neurons. This was in consonance with the delayed appearance of the 4R tau and a consequent delay in differentiation and impaired mitochondrial transport in cultured induced pluripotent stem cell (iPSc)-derived neurons from a GSS patient carrying the Y218N *PRNP* mutation [44], which could be due, among other factors, to the lack of PrP^C^ function. The same report included a decrease in total levels and increased phosphorylation of tau of neurons affected by *PRNP* mutation after 45 *DIV* [44]. Although we did not analyze the phosphorylation of tau in any of our embryonic/adult mutant mouse models in the present study, our results showed lower expression of tau when PrP^C^ was missing in adult mice brains. However, no changes in total tau levels were detected in primary cultures from ZH3 at 7 *DIV* (data not shown), in correlation with reported results in 7 *DIV* primary culture neurons with different PrP^C^ dosage with the use of a knock-out mouse model, primary cultures from *Prnp*^+/+^ embryos treated with siRNA-PrP^C^, or the Tg20 *Prnp* overexpressing model [42]. In addition, the *MAPT* analysis of a previous microarray using neuroblastoma N2a cells reinforced these results showing constant tau levels in this cell line independently of PrP^C^ expression [76]. This work suggested that PrP^C^ might be acting as a fine-tuning modulator of gene expression, as a post transcriptomic rather than transcriptomic regulator. This suggests that *MAPT* splicing is the first consequence of PrP^C^ ablation in differentiating neurons, while the consequence in mature neurons including adult mice brain reflects a misbalance in splicing forms, which results in a deficient tau turnover and an increase in susceptibility to hyperphosphorylation.

Concerning GSK3β, our results showed an increase in its activity parallel to lack of PrP^C^ expression, both in the brain extracts of the four mouse models analyzed and in primary cultures, correlating with changes in tau splicing. In fact, GSK3β, constitutively active in cells, is mostly regulated through inhibition by phosphorylation of ser9 [61]. In this sense, and as indicated above, PrP^C^ is one of the upstream GSK3β inhibitors in neurons [36]. Thus, a reduction of PrP^C^ function may affect increasing kinase activity parallel to an increase in 3R/4R tau ratio, as demonstrated by the reversion of the 3R/4R tau ratio using the GSK3β inhibitor SB-216763 in *Prnp*^0/0^ primary cultures. Of interest, dysfunction of the PI3K–Akt–GSK3 pathway is a common feature in cell cultures and in vivo models of prion disease [77,78,79]. These works reported that PrP_106–126_ peptide, widely used as a model of prion disease, as well as prion-activated GSK3β, induced as a consequence an increase in tau phosphorylation. In addition, changes in tau metabolism have been widely reported in prion disease models or prionopathies that present a loss of function of endogenous PrP^C^ in parallel to toxic effects of PrP^Sc^ [80,81,82,83,84]. It would be of interest to analyze the forms of tau splicing in all these scenarios. 

### 3.2. GSK3β Activity, Correlative with PrP^C^ Levels, Is Not Mandatory for 3R/4R Tau Ratio in AD

GSK3 kinase has been widely reported to be one of the kinases implicated in hyperphosphorylation of tau in AD [85] (reviewed in [86]). In addition, enhanced GSK3 activity has been found in the frontal cortex (FC) of AD samples [87] and its expression is upregulated in the hippocampus of AD patients [88]. However, related works focused on advanced levels of the disease, from Braak stage III to VI, corresponding to patients presenting with intermediate or severe dementia in clinicopathological correlation studies. Consequently, Braak stages I–II (from asymptomatic patients) were considered as controls [89,90]. As no previous study has considered the impact of changes in PrP^C^ expression and its correlation with GSK3β activity, we analyzed these parameters grouping human samples into Initial (I–II stages), Intermediate (III–IV stages), and Late (V–VI stages) considering the gradual expression of PrP^C^ in each of these. In this sense, our results showed a tendency to decrease GSK3β activity in samples with significant overexpression of PrP^C^ (both with WB and RT-PCR, from Braak stages I–II) in contrast to a progressive increase in GSK3β activity in advanced AD stages that reverse correlated with the progressive decrease of PrP^C^ levels. Our results also revealed a tendency to decrease the 3R/4R tau ratio parallel to the decreased GSK3β activity in samples with an increase in PrP^C^ levels (Braak I–II stages), while no reverse correlation between the 3R/4R tau ratio and PrP^C^ levels was found in samples with advanced disease that showed a recovery of GSK3β activity.

AD is considered a tauopathy with equal amounts of 3R and 4R tau splicing isoforms [91]. In fact, [45] reported that the same amount of all six isoforms was found in AD patients. However these studies considered the samples from Braak I–II stages as controls, as previously indicated for GSK3 [87,88]. Moreover, a regional isoform transition from 4R to 3R according to progression of the disease has been described [92]. Therefore, it is plausible that overexpression of PrP^C^ in the first stages of the disease could be affecting GSK3β activity and tau splicing, although other factors may be involved in parallel. In this sense, the loss of PrP^C^ expression in advanced disease may correlate with the cytological evolution of individual neurons: pretangle neurons (more 4R immunoreactivity) to NFTs (equal amount of 3R and 4R immunoreactivity) and then to ghost tangles (more 3R) [92]. In fact, splicing of human exon 10 responds to a complex regulation influenced by cell type, the identity of its flanking exons, and sequences within and near the exon itself and several splicing regulators [93]. In addition, exon tau splicing is influenced by the extent of flanking introns and by additional cis sequences [94]. Between the multiple factors implicated in *MAPT* splicing [19], noncoding RNAs are considered of interest in the onset and progression of AD [95]. Among these, miR132-3p, with effects on tau exon 10 exclusion [96], has been previously reported as downregulated in the progression of AD [48,49]. Thus, although our results did not reach statistical significance, probably due to the small number of samples, we confirmed the progressive downregulation of miR132-3p in AD samples grouped into Initial-Intermediate-Late but not significantly different in adult mouse brains with a different dosage of PrP^C^ expression, which would explain how additional splicing factors (for instance miR132-3p) reduce the effects of PrP^C^-GSK3β in the prevalence of 3R and 4R tau isoforms in AD.

Lastly, in the effort to understand the relationship of PrP^C^ and tau, we recently described the transcriptional control of *PRNP* promoter by tau, which in turn may explain the overexpression of PrP^C^ in Initial stages of AD [41], but the biological meaning of this is unknown despite previous data concerning the role of PrP^C^ in the control of tau expression. Thus, the goal of the present study was to show the implication of PrP^C^ through GSK3β activity in alternative splicing of tau exon 10, both in mice and humans. Although the repercussions of this finding in AD progression are also determined by additional splicing factors, it is of interest to analyze the potential of PrP^C^ in control of tau exon 10 splicing in other tauopathies.

## 4. Materials and Methods

### 4.1. Human Hippocampal Samples

Human cases comprised 13 non-AD (nAD in Table 1) and 57 AD postmortem brains from the HUB-ICO-IDIBELL Biobank. Basic patient data are shown in Table 1. Cases with and without clinical neurological disease were processed in the same way following the same sampling and staining protocols. At autopsy, half of each brain was fixed in 10% buffered formalin, while the other half was cut in coronal sections 1 cm thick, frozen on dry ice, and stored at −80 °C until use. In addition, 2 mm thick samples of the cerebral isocortex, cingulum, hippocampus and entorhinal cortex, and brainstem were fixed with phosphate buffered 4% paraformaldehyde for 24 h, cryoprotected with 30% sucrose, frozen on dry ice, and stored at −80 °C until use.

Following neuropathological examination, AD cases were categorized as stages I to VI of Braak and Braak [97,98]. Healthy cases (non-AD) did not show neurological or metabolic disease, and the neuropathological examination, carried out in similar regions and with the same methods as in AD cases, did not show lesions. In particular, no amyloid or tau deposits were seen in the regions examined. Samples were analyzed unblinded to Braak stage.

### 4.2. Mouse Strains and Genotyping

We used adult male mice at 3 months of age from the four mouse lines described below.

ZH1 *Prnp*^0/0^ mice, purchased from The European Mouse Mutant Archive (EMMA), were generated in a mixed genetic background: C57BL/6J × 129/Sv(ev) [99]. Specific primers to *Prnp* genotyping were designed in our laboratory based on the original P3 and P10 primers described by [99]: neo: 5′-gccttctatcgccttcttgac-3′; 3′NCnew: 5′-gctacaggtggataacccctc-3′ and P10new: 5′-cataatcagtggaacaagccc-3′. The 40 cycling conditions were 45” 95 °C, 45″ 62 °C, and 1′ 72 °C, followed by a final extension at 72 ° C for 5 min. 

Co-isogenic C57BL/6J-*Prnp*^0/0^ mice (Zürich 3, ZH3; a gift from Adriano Aguzzi) were generated as described [67]. Specific primers to *Prnp* genotyping were Zh3 for: 5′-agggttgacgccatgacttt-3′ and Zh3 rev: 5′-tatgggtaccccctccttgg-3′. The 35 cycling conditions were: 30″ 94 °C, 30″ 58 °C, and 45″ 72 °C, followed by a final extension at 72 °C for 5 min.

The TgTP6.3 mouse line is a transgenic mouse carrying a tau-tagged green fluorescence protein (GFP) transgene generated by [100]. This line was maintained as heterozygotes for the tau-GFP transgene, and adult mice carrying the transgene were identified by fluorescence microscopy of ear tissue obtained as a by-product of ear clipping for identification purposes. 

P301S mice transgenic for the human tau gene on a B6C3F1 background [101] were used. This mouse model carries a mutant (P301S) human *MAPT* gene encoding T34-tau isoform (1N4R); it is driven by the mouse prion-protein promoter (*Prnp*) on a B6C3H/F1 genetic background. These mice were purchased from Jackson Laboratory. The transgene was detected by using specific primers: 5′-ggggacacgtctccacggcatctcagcaatgtctcc-3′ (301S for); 5′-tcccccagcctagaccacgagaat-3′ (301S rev); 5′-caaatgttgcttgtctggtg-3′ (301S int for); and 5′-gtcagtcgagtgcacagttt-3′ (301S int rev). The 35 cycling conditions were 30″ 94 °C, 1′ 57 °C, and 1′ 72 °C, followed by a final extension at 72 ° C for 5 min. 

In addition, embryos from pregnant females from ZH3-*Prnp*^0/0^ mice were also used. Females were crossed overnight, the mating day was considered to be embryonic day 0 (E0.5), and the offspring were genotyped. 

### 4.3. Primary Embryonic Cortical Cultures and Transfection

E15.5–16.5 mouse embryo brains were dissected and washed in ice-cold 0.1 M phosphate-buffered saline (PBS) containing 6.5 mg/mL glucose. The meninges were removed and the cortical lobes isolated. Tissue pieces were trypsinized for 15 min at 37 °C. After addition of horse serum and centrifugation, cells were dissociated by trituration in 0.1 M PBS containing 0.025% DNase with a polished glass pipette (all from Sigma-Aldrich, Darmstadt, Germany). Dissociated cells were plated at ~3000 cells/mm^2^ on plates (Nunc, Denmark) coated with poly-D-lysine (Sigma-Aldrich). The culture medium was Neurobasal^TM^ supplemented with 2 mM glutamine, 6.5 mg/mL glucose, antibiotics (Pen/Strept), 5% horse serum, and B27 (Invitrogen-Thermo Fisher Scientific, Waltham, MA, USA). After 72 h, 5 µM cytosine β-D-arabinofuranosidehydrochloride (AraC) (Sigma-Aldrich) was added for 48 h to inhibit the growth of dividing non-neuronal cells. Cultures were used after at least 7 days in vitro.

For PrP^C^ overexpression on cortical cultures, PrP^C^-encoding plasmid (pcDNA 3.1 backbone), provided by D. A. Harris (Boston University School of Medicine, Boston, MA, USA) was transfected using Lipofectamine 2000 (Invitrogen-Thermo Fisher Scientific), according to the manufacturer’s instructions.

### 4.4. Microfluidic Devices

One microfluidic device was used in an optimized modification of our previous design of large dual-chamber, open neuronal co-culture, and of designs reported by [102]. The open microfluidic device consists of two main open chambers interconnected by 100 microchannels. The large chamber areas (9 mm × 16 mm) facilitate effective cell culture and easy handling. The small cross-section areas of microchannels (10 µm × 10 µm) restrict the crossing of cortical neuron cell bodies but permit the passage of neuronal processes. The microfluidic device was made of poly(dimethylsiloxane) (PDMS) using standard photolithography and soft lithography.

### 4.5. Western Blot Analysis

Soluble extract from human hippocampal samples, mouse cortex brains, and cultured cells was processed for WB. The collected samples were homogenized in lysis buffer: 50 mM Hepes, 150 mM NaCl, 1.5 mM MgCl_2_, 1 mM EGTA, 10% glycerol, 1% and Triton X-100 with supplemental 1× protease inhibitor cocktail (Roche Diagnostic, Basel, Switzerland) and 1 µM okadaic acid (Merck Millipore, Burlington, MA, USA), 0.1 M sodium fluoride, 10 mM sodium pyrophosphate, and 200 µM sodium orthovanadate (Sigma-Aldrich) as phosphatase inhibitor. After this, samples were centrifuged at 13,000× *g* for 20 min at 4 °C. The resulting supernatant was normalized for protein content using BCA kit (Pierce Biotechnology, Waltham, MA, US). Cell extracts were boiled in Laemmli sample buffer [103] at 96 °C for 5 min, followed by 10% SDS-PAGE electrophoresis, and electrotransferred to nitrocellulose membranes for 1 h at 4 °C. Membranes were blocked with 5% non-fat milk in 0.1 M Tris-buffered saline (pH 7.4) for 1 h and incubated overnight in a solution containing primary antibodies. After incubation with peroxidase-tagged secondary antibodies (1:2000 diluted), membranes were revealed with the ECL-plus chemiluminescence WB kit (Amersham-GE Healthcare, Amersham, UK).

In our experiments, the same amount of protein was loaded per lane (15 µg). In addition, levels of total, 3R and 4R tau were normalized to actin, while levels of phospho-GSK3β tyr^279/216^ and phospho-GSK3β ser^9^ were normalized to total GSK3β protein. To do this, some nitrocellulose membranes were used to detect two antigens in parallel (total tau/actin and 4R tau/actin respectively), while additional membranes were used to detect consecutive antigens. To perform this sequential incubation, membranes were incubated in 25 mL of stripping solution (2% SDS, 62.5 mM Tris pH 6.8 and 100 mM 2-mercaptoethanol) for 30 min at 65 °C and then extensively washed before re-incubation with blocking buffer and antibodies for re-blotting. 

For the quantification, developed films were scanned at 2400 × 2400 dpi (i800 MICROTEK high quality film scanner), and the densitometric analysis was performed using ImageJ^TM^ software. 

### 4.6. Immunohistochemical Procedures

For immunohistochemistry of postnatal mice, the animals were anesthetized with ketamine (35 mg/kg) and xylazine (2 mg/kg) (Sigma-Aldrich) and perfused with 4% paraformaldehyde (PFA) in 0.1 M PBS pH 7.4. After perfusion, brains were removed and post-fixed overnight in the same fixative solution, cryoprotected in 30% sucrose in 0.1 M PBS, sectioned (30 µm) on a freezing microtome (Leica, Wetzlar, Germany), and processed. Briefly, free-floating sections were rinsed in 0.1M PBS, and the endogenous peroxidase activity was blocked by incubation in 3% hydrogen peroxide (H_2_O_2_) and 10% methanol dissolved in 0.1 M PBS. After extensive rinsing, sections were incubated in 0.1 M PBS containing 0.2% gelatin, 10% normal goat serum, 0.2% glycine, and 0.5% Triton-X 100 for 2 h at room temperature. Afterwards, sections were incubated overnight at 4 °C with the primary antibody. Afterwards, sections were incubated with secondary biotinylated antibodies (2 h, 1:200 diluted) and streptavidin-horseradish peroxidase complex (2 h, 1:400 diluted), both at room temperature. Peroxidase activity was revealed with 0.03% diaminobenzidine (DAB) and 0.002% H_2_O_2_. After rinsing, sections were mounted onto gelatinized slides and dehydrated, cleared in xylol, and coverslipped with Eukitt^TM^ (Merck Millipore). Immunohistochemical controls, including omission of the primary antibody or its replacement by normal serum, were devoid of staining. 

Photomicrographs were obtained using an Olympus BX61 microscope equipped with a cooled digital DP72L camera. 

### 4.7. RT-qPCR

Total RNA from human hippocampal samples, mouse cortex brains, and cultured cells was extracted with mirVana’s isolation kit (Ambion, Austin, TX, USA) following the manufacturer’s instructions. Total purified RNAs were used to generate the corresponding cDNAs, which served as PCR templates for PCR assays. 

Quantitative reverse transcription PCR (RT-qPCR) for mRNAs was performed in triplicate using the following primers: (5′-ccccctaagtcaccatcagctagt-3′) and (5′-cactttgctcaggtccaccggc-3′) for mouse tau [66], (5′-acccaagtcgccgtcttccgcc-3′) and (5′-caccttgctcaggtcaactggt-3′) for human tau, (5′-gtcaggtcgaagattggctctact-3′) and (5′-gcttgtagactatttgcaccttgc-3′) for mouse 3R tau, (5′-tgtcaggtcgaagattggctc-3′) and (5′-cttattattatctgcaccttgccac-3′) for mouse 4R tau, (5′-gaagaatgtcaagtccaagatcgg-3′) and (5′-gcttgtagactatttgcaccttgc-3′) for human 3R tau, (5′-ggtgcagataattaataagaagctgga-3′) and (5′-gtgtttgatattatcctttgagccac-3′) for human 4R tau and (5′-agtcgttgccaaaatggatca-3’) and (5′-aaaaaccaacctcaagcatgtgg-3′) for PrP^C^ [104]. PCR amplification and detection were performed with the ROCHE LightCycler 480 detector, using 2× SYBR GREEN Master Mix (Roche Diagnostic, Switzerland) as reagent, following the manufacturer’s instructions. The reaction profile was denaturation-activation cycle (95 °C for 10 min) followed by 40 cycles of denaturation-annealing-extension (95 °C for 10 s, 55 °C for 15 s and 72 °C for 20 s). mRNA levels were calculated using the LightCycler 480 software. Data were analyzed with SDS 1.9.1 Software (Applied Biosystems, USA) following the 2^−∆∆CT^ method of Applied Biosystems [105]. The results were normalized for the expression levels of the housekeeping gene, (5′-aggtcggtgtgaacggatttg-3′) and (5′-tgtagaccatgtagttgaggtca-3′) for murine *gapdh* or (5′-tccaaaatcaagtggggcga-3′) and (5′-tctccatggtggtgaagacg-3′) for human *GAPDH*, which were quantified simultaneously with the target gene [106].

RT-qPCR for miR132-3p was performed using the miRCURY LNA™ miRNA PCR System and using the hsa-miR-132-3p, LNA™ PCR primer set (Exiqon, Copenhagen, Denmark). PCR amplification and detection were performed with the Roche LightCycler 480 detector, using 2× SYBR GREEN Master Mix. The reaction profile was polymerase activation/denaturation (95 °C for 10 min) followed by 40 amplification cycles (95 °C for 10 s, 60 °C for 20 s). miRNA levels were calculated using the LightCycler^TM^ 480 software. Samples were normalized for the relative expression of the housekeeping small nuclear RNAs U6 (U6 snRNA LNA™ PCR primer set, Exiqon) and miR103a-3p (hsa-miR-103a-3p, LNA™ PCR primer set, Exiqon). Housekeeping genes showed no variability between analyzed groups.

### 4.8. Antibodies and Reagents

Several antibodies were used for immunohistochemistry and western blot. Monoclonal anti-PrP 6H4 (Prionics, Zurich, Switzerland) (WB, 1:5000) was used to determine PrP^C^ levels. Total levels of the tau protein were checked with monoclonal tau 5 antibody (IHC/WB, 1:200/1:1000 diluted respectively, Invitrogen-Thermo Fisher Scientific). The different tau isoforms 3R and 4R were checked with monoclonal RD3 (IHC/WB, 1:200/1:1000) and RD4 (WB, 1:1000) antibodies respectively (Merck Millipore). GSK3 (clone 4G-1E, WB, 1:3000), GSK3 phospho-tyr^279/216^ (clone 5G-2F, WB, 1:2000) and GSK3 phospho-ser^9^ (clone 2D3, WB, 1:250) were from Upstate Biotechnology, Lake Placid, NY, USA. The monoclonal anti-β-actin antibody (Merck Millipore), diluted 1:20,000, was used as internal control in WBs. 

The GSK3β inhibitor SB-216763 was purchased from Sigma-Aldrich and was used at a concentration of 3 µM.

### 4.9. Statistical Processing 

Data analysis was performed using Prism 6.0 (GraphPad Software). Data were obtained using *t*-test. Differences with * *p* < 0.05, ** *p* < 0.01, and *** *p* < 0.001, **** p < 0.0001 were considered significant. All data are expressed as mean ± standard error of the mean (S.E.M.).

## Figures and Tables

**Table 1:** Control (nAD) and AD samples, categorized as stages I to VI of Braak and Braak, used in this study.

Case Number	Braak and Braak Stage	Gender	Age	Post-Mortem Delay	Analysis
nAD1	-	M	39	9 h 15 min	WB
nAD2	-	F	46	14 h 15 min	WB
nAD3	-	M	53	7 h 25 min	WB
nAD4	-	M	46	15 h	WB
nAD5	-	M	43	4 h 35 min	WB/RT-PCR
nAD6	-	M	52	3 h	WB
nAD7	-	M	51	3 h 30 min	WB
nAD8	-	F	86	4 h	WB
nAD9	-	F	46	9 h 35 min	RT-PCR
nAD10	-	M	70	13 h	RT-PCR
nAD11	-	F	82	11 h	RT-PCR
nAD12	-	F	46	20 h	RT-PCR
nAD13	-	M	61	2 h 45 min	RT-PCR
AD1	I	M	61	3 h 40 min	WB
AD2	I	M	53	6 h 15 min	WB
AD3	I	M	74	4 h	WB
AD4	I	M	71	11 h 30 min	WB
AD5	I	M	64	2 h 15 min	WB
AD6	I	F	79	3 h 35 min	WB/RT-PCR
AD7	I	M	65	5 h 15 min	WB
AD8	I	F	75	4 h 55 min	WB
AD9	I	M	63	6 h	WB
AD10	I	M	68	10 h 55 min	WB
AD11	I	M	64	8 h 35 min	RT-PCR
AD12	I	M	61	5 h 35 min	RT-PCR
AD13	I	M	67	14 h 40 min	RT-PCR
AD14	I	F	73	15 h 45 min	RT-PCR
AD15	I	M	70	5 h	RT-PCR
AD16	II	M	65	5 h	RT-PCR
AD17	II	F	77	11 h	WB
AD18	II	M	65	5 h	WB
AD19	II	M	66	4 h 55 min	WB
AD20	II	M	72	8 h 45 min	WB
AD21	II	M	71	5 h 15 min	WB
AD22	II	M	66	5 h	WB
AD23	II	F	60	9 h 40 min	WB
AD24	II	F	80	3 h 30 min	WB
AD25	II	F	75	4 h 55 min	RT-PCR
AD26	II	F	86	4 h 15 min	RT-PCR
AD27	II	M	55	9 h 45 min	RT-PCR
AD28	II	F	57	4 h 30 min	RT-PCR
AD29	II	M	69	3 h 45 min	WB
AD30	II	M	74	5 h 30 min	WB/RT-PCR
AD31	II	M	86	5 h 35 min	WB
AD32	III	F	81	1 h 30 min	WB
AD33	III	F	71	7 h 15 min	WB/RT-PCR
AD34	III	F	77	11 h 30 min	WB/RT-PCR
AD35	III	F	67	6 h 10 min	WB
AD36	III	M	69	13 h 10 min	WB
AD37	III	F	83	2 h 30 min	RT-PCR
AD38	III	M	87	3 h 30 min	RT-PCR
AD39	III	F	82	4 h 50 min	RT-PCR
AD40	III	M	64	6 h	WB/RT-PCR
AD41	IV	F	80	2 h 45 min	WB
AD42	IV	F	81	12 h	WB
AD43	IV	M	84	12 h 45 min	WB
AD44	IV	M	79	50 min	WB
AD45	IV	M	83	7 h 25 min	WB/RT-PCR
AD46	IV	F	90	10 h	WB/RT-PCR
AD47	IV	F	81	5 h	RT-PCR
AD48	V	M	87	7 h 5 min	WB/RT-PCR
AD49	V	M	75	11 h 30 min	WB/RT-PCR
AD50	V	M	82	3 h 45 min	WB/RT-PCR
AD51	V	M	77	16 h	WB
AD52	V	F	82	1 h 45 min	WB/RT-PCR
AD53	V	F	75	4 h 15 min	WB/RT-PCR
AD54	V	M	93	3 h	RT-PCR
AD55	VI	M	86	20 h 35 min	WB
AD56	VI	M	67	8 h	WB/RT-PCR
AD57	VI	F	56	7 h	WB/RT-PCR

F: female; M: male; nAD: non-AD.

## Data Availability

Not applicable.

## Supplementary Materials

The following are available online at https://www.mdpi.com/article/10.3390/ijms22105370/s1, Figure S1: Changes in GSK3β activity after PrP^C^ overexpression in *Prnp*^+/+^ neural primary cultures or using SB-216763 inhibitor in ZH3 mice cultured neurons; Figure S2: GSK3β activity analyzed with WB of hippocampal necropsies from AD patients compared to healthy cases; Figure S3: miR132-3p expression in human AD and mouse samples.

## Abbreviations

Aβ	β-amyloid
AD	Alzheimer’s disease
ADDLs	Aβ-derived diffusible ligands
CBD	Corticobasal degeneration
CNS	Central nervous system
CTCF	Corrected total cell fluorescence
DIV	Days in vitro
FTDP-17	Frontotemporal dementia and parkinsonism linked to chromosome 17
GFP	Green fluorescence protein
GR	Glutathione reductase
GSK3β	Glycogen Synthase Kinase 3-β
GSS	Gerstmann-Sträussler-Scheinker syndrome
iPSCs	Induced pluripotent stem cells
LTP	Long term potentiation
MAPT	Microtubule-associated protein tau
MT	Microtubules
NFT	Neurofibrillary tangles
PHF,	Paired helical filaments
PrP^C^	Cellular prion protein
PrP^SC^	Scrapie isoform of PrP^C^
PD	Pick’s disease
PSP	Progressive supranuclear palsy
SOD	Superoxide dismutase
STI-1	Stress-inducible protein 1
TSEs	Transmissible spongiform encephalopathies
WT	Wild type

## References

[1] Avila J. (2000). Tau aggregation into fibrillar polymers: Tauopathies. FEBS Lett..

[2] Kovacs G.G. (2015). Invited review: Neuropathology of tauopathies: Principles and practice. Neuropathol. Appl. Neurobiol..

[3] Lee V.M., Goedert M., Trojanowski J.Q. (2001). Neurodegenerative tauopathies. Annu. Rev. Neurosci..

[4] Buee L., Delacourte A. (1999). Comparative biochemistry of tau in progressive supranuclear palsy, corticobasal degeneration, FTDP-17 and Pick’s disease. Brain Pathol..

[5] Lowe J., Kalaria R., Love S., Budka H., Ironside J., Perry A. (2015). Dementia. Greenfield’s Neuropathology.

[6] Selkoe D.J. (2001). Alzheimer’s disease: Genes, proteins, and therapy. Physiol. Rev..

[7] Hardy J.A., Higgins G.A. (1992). Alzheimer’s disease: The amyloid cascade hypothesis. Science.

[8] Hardy J. (2003). The relationship between amyloid and tau. J. Mol. Neurosci..

[9] Braak H., Braak E. (1991). Neuropathological stageing of Alzheimer-related changes. Acta Neuropathol..

[10] Iqbal K., Liu F., Gong C.X., Grundke-Iqbal I. (2010). Tau in Alzheimer disease and related tauopathies. Curr. Alzheimer Res..

[11] Pigino G., Morfini G., Atagi Y., Deshpande A., Yu C., Jungbauer L., LaDu M., Busciglio J., Brady S. (2009). Disruption of fast axonal transport is a pathogenic mechanism for intraneuronal amyloid beta. Proc. Natl. Acad. Sci. USA.

[12] Lu M., Kosik K.S. (2001). Competition for microtubule-binding with dual expression of tau missense and splice isoforms. Mol. Biol. Cell.

[13] Sennvik K., Boekhoorn K., Lasrado R., Terwel D., Verhaeghe S., Korr H., Schmitz C., Tomiyama T., Mori H., Krugers H. (2007). Tau-4R suppresses proliferation and promotes neuronal differentiation in the hippocampus of tau knockin/knockout mice. FASEB J..

[14] Avila J., Lucas J.J., Perez M., Hernandez F. (2004). Role of tau protein in both physiological and pathological conditions. Physiol. Rev..

[15] Goedert M., Spillantini M.G., Jakes R., Rutherford D., Crowther R.A. (1989). Multiple isoforms of human microtubule-associated protein tau: Sequences and localization in neurofibrillary tangles of Alzheimer’s disease. Neuron.

[16] Lewis J., McGowan E., Rockwood J., Melrose H., Nacharaju P., van Slegtenhorst M., Gwinn-Hardy K., Paul Murphy M., Baker M., Yu X. (2000). Neurofibrillary tangles, amyotrophy and progressive motor disturbance in mice expressing mutant (P301L) tau protein. Nat. Genet..

[17] Liu F., Gong C.X. (2008). Tau exon 10 alternative splicing and tauopathies. Mol. Neurodegener..

[18] Dickson D.W., Kouri N., Murray M.E., Josephs K.A. (2011). Neuropathology of frontotemporal lobar degeneration-tau (FTLD-tau). J. Mol. Neurosci..

[19] Park S.A., Ahn S.I., Gallo J.M. (2016). Tau mis-splicing in the pathogenesis of neurodegenerative disorders. BMB Rep..

[20] Hanger D.P., Byers H.L., Wray S., Leung K.Y., Saxton M.J., Seereeram A., Reynolds C.H., Ward M.A., Anderton B.H. (2007). Novel phosphorylation sites in tau from Alzheimer brain support a role for casein kinase 1 in disease pathogenesis. J. Biol. Chem..

[21] Chen K.L., Yuan R.Y., Hu C.J., Hsu C.Y. (2010). Amyloid-beta peptide alteration of tau exon-10 splicing via the GSK3beta-SC35 pathway. Neurobiol. Dis..

[22] Moleres F.J., Velayos J.L. (2005). Expression of PrP(C) in the rat brain and characterization of a subset of cortical neurons. Brain Res..

[23] Ford M.J., Burton L.J., Morris R.J., Hall S.M. (2002). Selective expression of prion protein in peripheral tissues of the adult mouse. Neuroscience.

[24] Moser M., Colello R.J., Pott U., Oesch B. (1995). Developmental expression of the prion protein gene in glial cells. Neuron.

[25] Prusiner S.B. (1982). Novel proteinaceous infectious particles cause scrapie. Science.

[26] Chiarini L.B., Freitas A.R., Zanata S.M., Brentani R.R., Martins V.R., Linden R. (2002). Cellular prion protein transduces neuroprotective signals. EMBO J..

[27] Zanata S.M., Lopes M.H., Mercadante A.F., Hajj G.N., Chiarini L.B., Nomizo R., Freitas A.R., Cabral A.L., Lee K.S., Juliano M.A. (2002). Stress-inducible protein 1 is a cell surface ligand for cellular prion that triggers neuroprotection. EMBO J..

[28] Rangel A., Burgaya F., Gavin R., Soriano E., Aguzzi A., Del Rio J.A. (2007). Enhanced susceptibility of Prnp-deficient mice to kainate-induced seizures, neuronal apoptosis, and death: Role of AMPA/kainate receptors. J. Neurosci. Res..

[29] Brown D.R., Nicholas R.S., Canevari L. (2002). Lack of prion protein expression results in a neuronal phenotype sensitive to stress. J. Neurosci. Res..

[30] Sakudo A., Lee D.C., Saeki K., Nakamura Y., Inoue K., Matsumoto Y., Itohara S., Onodera T. (2003). Impairment of superoxide dismutase activation by N-terminally truncated prion protein (PrP) in PrP-deficient neuronal cell line. Biochem. Biophys. Res. Commun..

[31] White A.R., Collins S.J., Maher F., Jobling M.F., Stewart L.R., Thyer J.M., Beyreuther K., Masters C.L., Cappai R. (1999). Prion protein-deficient neurons reveal lower glutathione reductase activity and increased susceptibility to hydrogen peroxide toxicity. Am. J. Pathol..

[32] Lee Y.J., Baskakov I.V. (2013). The cellular form of the prion protein is involved in controlling cell cycle dynamics, self-renewal, and the fate of human embryonic stem cell differentiation. J. Neurochem..

[33] Steele A.D., Emsley J.G., Ozdinler P.H., Lindquist S., Macklis J.D. (2006). Prion protein (PrP^c^) positively regulates neural precursor proliferation during developmental and adult mammalian neurogenesis. Proc. Natl. Acad. Sci. USA.

[34] Loubet D., Dakowski C., Pietri M., Pradines E., Bernard S., Callebert J., Ardila-Osorio H., Mouillet-Richard S., Launay J.M., Kellermann O. (2012). Neuritogenesis: The prion protein controls beta1 integrin signaling activity. FASEB J..

[35] Gao L., Zhao M., Ye W., Huang J., Chu J., Yan S., Wang C., Zeng R. (2016). Inhibition of glycogen synthase kinase-3 (GSK3) promotes the neural differentiation of full-term amniotic fluid-derived stem cells towards neural progenitor cells. Tissue Cell.

[36] Hernandez-Rapp J., Martin-Lanneree S., Hirsch T.Z., Pradines E., Alleaume-Butaux A., Schneider B., Baudry A., Launay J.M., Mouillet-Richard S. (2014). A PrP(C)-caveolin-Lyn complex negatively controls neuronal GSK3beta and serotonin 1B receptor. Sci. Rep..

[37] Whitehouse I.J., Miners J.S., Glennon E.B., Kehoe P.G., Love S., Kellett K.A., Hooper N.M. (2013). Prion protein is decreased in Alzheimer’s brain and inversely correlates with BACE1 activity, amyloid-beta levels and Braak stage. PLoS ONE.

[38] Lauren J., Gimbel D.A., Nygaard H.B., Gilbert J.W., Strittmatter S.M. (2009). Cellular prion protein mediates impairment of synaptic plasticity by amyloid-beta oligomers. Nature.

[39] Younan N.D., Chen K.F., Rose R.S., Crowther D.C., Viles J.H. (2018). Prion protein stabilizes amyloid-beta (Abeta) oligomers and enhances Abeta neurotoxicity in a Drosophila model of Alzheimer’s disease. J. Biol. Chem..

[40] Calella A.M., Farinelli M., Nuvolone M., Mirante O., Moos R., Falsig J., Mansuy I.M., Aguzzi A. (2010). Prion protein and Abeta-related synaptic toxicity impairment. EMBO Mol. Med..

[41] Lidon L., Vergara C., Ferrer I., Hernandez F., Avila J., del Rio J.A., Gavin R. (2020). Tau Protein as a New Regulator of Cellular Prion Protein Transcription. Mol. Neurobiol..

[42] Vergara C., Ordonez-Gutierrez L., Wandosell F., Ferrer I., del Rio J.A., Gavin R. (2015). Role of PrP(C) Expression in Tau Protein Levels and Phosphorylation in Alzheimer’s Disease Evolution. Mol. Neurobiol..

[43] Hernandez F., Perez M., Lucas J.J., Mata A.M., Bhat R., Avila J. (2004). Glycogen synthase kinase-3 plays a crucial role in tau exon 10 splicing and intranuclear distribution of SC35. Implications for Alzheimer’s disease. J. Biol. Chem..

[44] Matamoros-Angles A., Gayosso L.M., Richaud-Patin Y., di Domenico A., Vergara C., Hervera A., Sousa A., Fernandez-Borges N., Consiglio A., Gavin R. (2017). iPS Cell Cultures from a Gerstmann-Straussler-Scheinker Patient with the Y218N PRNP Mutation Recapitulate tau Pathology. Mol. Neurobiol..

[45] Boutajangout A., Boom A., Leroy K., Brion J.P. (2004). Expression of tau mRNA and soluble tau isoforms in affected and non-affected brain areas in Alzheimer’s disease. FEBS Lett..

[46] Chambers C.B., Lee J.M., Troncoso J.C., Reich S., Muma N.A. (1999). Overexpression of four-repeat tau mRNA isoforms in progressive supranuclear palsy but not in Alzheimer’s disease. Ann. Neurol..

[47] Schmitz M., Wulf K., Signore S.C., Schulz-Schaeffer W.J., Kermer P., Bahr M., Wouters F.S., Zafar S., Zerr I. (2014). Impact of the Cellular Prion Protein on Amyloid-beta and 3PO-Tau Processing. J. Alzheimers Dis..

[48] Lau P., Bossers K., Janky R., Salta E., Frigerio C.S., Barbash S., Rothman R., Sierksma A.S., Thathiah A., Greenberg D. (2013). Alteration of the microRNA network during the progression of Alzheimer’s disease. EMBO Mol. Med..

[49] Pichler S., Gu W., Hartl D., Gasparoni G., Leidinger P., Keller A., Meese E., Mayhaus M., Hampel H., Riemenschneider M. (2017). The miRNome of Alzheimer’s disease: Consistent downregulation of the miR-132/212 cluster. Neurobiol. Aging.

[50] Goedert M., Spillantini M.G., Potier M.C., Ulrich J., Crowther R.A. (1989). Cloning and sequencing of the cDNA encoding an isoform of microtubule-associated protein tau containing four tandem repeats: Differential expression of tau protein mRNAs in human brain. EMBO J..

[51] Hefti M.M., Farrell K., Kim S., Bowles K.R., Fowkes M.E., Raj T., Crary J.F. (2018). High-resolution temporal and regional mapping of MAPT expression and splicing in human brain development. PLoS ONE.

[52] Sposito T., Preza E., Mahoney C.J., Seto-Salvia N., Ryan N.S., Morris H.R., Arber C., Devine M.J., Houlden H., Warner T.T. (2015). Developmental regulation of tau splicing is disrupted in stem cell-derived neurons from frontotemporal dementia patients with the 10 + 16 splice-site mutation in MAPT. Hum. Mol. Genet..

[53] Niblock M., Gallo J.M. (2012). Tau alternative splicing in familial and sporadic tauopathies. Biochem. Soc. Trans..

[54] Linden R., Martins V.R., Prado M.A., Cammarota M., Izquierdo I., Brentani R.R. (2008). Physiology of the prion protein. Physiol. Rev..

[55] Shi F., Yang Y., Wang T., Kouadir M., Zhao D., Hu S. (2016). Cellular Prion Protein Promotes Neuronal Differentiation of Adipose-Derived Stem Cells by Upregulating miRNA-124. J. Mol. Neurosci..

[56] Pantera B., Bini C., Cirri P., Paoli P., Camici G., Manao G., Caselli A. (2009). PrP^c^ activation induces neurite outgrowth and differentiation in PC12 cells: Role for caveolin-1 in the signal transduction pathway. J. Neurochem..

[57] Hooper C., Killick R., Lovestone S. (2008). The GSK3 hypothesis of Alzheimer’s disease. J. Neurochem..

[58] Griebel G., Stemmelin J., Lopez-Grancha M., Boulay D., Boquet G., Slowinski F., Pichat P., Beeske S., Tanaka S., Mori A. (2019). The selective GSK3 inhibitor, SAR502250, displays neuroprotective activity and attenuates behavioral impairments in models of neuropsychiatric symptoms of Alzheimer’s disease in rodents. Sci. Rep..

[59] Alvarez G., Munoz-Montano J.R., Satrustegui J., Avila J., Bogonez E., Diaz-Nido J. (1999). Lithium protects cultured neurons against beta-amyloid-induced neurodegeneration. FEBS Lett..

[60] Koehler D., Shah Z.A., Williams F.E. (2019). The GSK3beta inhibitor, TDZD-8, rescues cognition in a zebrafish model of okadaic acid-induced Alzheimer’s disease. Neurochem. Int..

[61] Llorens-Martin M., Jurado J., Hernandez F., Avila J. (2014). GSK-3beta, a pivotal kinase in Alzheimer disease. Front. Mol. Neurosci..

[62] Saraswati A.P., Ali Hussaini S.M., Krishna N.H., Babu B.N., Kamal A. (2018). Glycogen synthase kinase-3 and its inhibitors: Potential target for various therapeutic conditions. Eur. J. Med. Chem..

[63] Bradley C.A., Peineau S., Taghibiglou C., Nicolas C.S., Whitcomb D.J., Bortolotto Z.A., Kaang B.K., Cho K., Wang Y.T., Collingridge G.L. (2012). A pivotal role of GSK-3 in synaptic plasticity. Front. Mol. Neurosci..

[64] Ostapchenko V.G., Beraldo F.H., Mohammad A.H., Xie Y.F., Hirata P.H., Magalhaes A.C., Lamour G., Li H., Maciejewski A., Belrose J.C. (2013). The prion protein ligand, stress-inducible phosphoprotein 1, regulates amyloid-beta oligomer toxicity. J. Neurosci..

[65] Kosik K.S., Orecchio L.D., Bakalis S., Neve R.L. (1989). Developmentally regulated expression of specific tau sequences. Neuron.

[66] McMillan P., Korvatska E., Poorkaj P., Evstafjeva Z., Robinson L., Greenup L., Leverenz J., Schellenberg G.D., D’Souza I. (2008). Tau isoform regulation is region- and cell-specific in mouse brain. J. Comp. Neurol..

[67] Nuvolone M., Hermann M., Sorce S., Russo G., Tiberi C., Schwarz P., Minikel E., Sanoudou D., Pelczar P., Aguzzi A. (2016). Strictly co-isogenic C57BL/6J-Prnp−/− mice: A rigorous resource for prion science. J. Exp. Med..

[68] Gustke N., Trinczek B., Biernat J., Mandelkow E.M., Mandelkow E. (1994). Domains of tau protein and interactions with microtubules. Biochemistry.

[69] Butner K.A., Kirschner M.W. (1991). Tau protein binds to microtubules through a flexible array of distributed weak sites. J. Cell Biol..

[70] Tarhan M.C., Orazov Y., Yokokawa R., Karsten S.L., Fujita H. (2013). Biosensing MAPs as “roadblocks”: Kinesin-based functional analysis of tau protein isoforms and mutants using suspended microtubules (sMTs). Lab Chip.

[71] Vershinin M., Xu J., Razafsky D.S., King S.J., Gross S.P. (2008). Tuning microtubule-based transport through filamentous MAPs: The problem of dynein. Traffic.

[72] Urrea L., Segura-Feliu M., Masuda-Suzukake M., Hervera A., Pedraz L., Aznar J.M., Vila M., Samitier J., Torrents E., Ferrer I. (2017). Involvement of Cellular Prion Protein in alpha-Synuclein Transport in Neurons. Mol. Neurobiol..

[73] Aulic S., Masperone L., Narkiewicz J., Isopi E., Bistaffa E., Ambrosetti E., Pastore B., de Cecco E., Scaini D., Zago P. (2017). alpha-Synuclein Amyloids Hijack Prion Protein to Gain Cell Entry, Facilitate Cell-to-Cell Spreading and Block Prion Replication. Sci. Rep..

[74] Schmitz M., Greis C., Ottis P., Silva C.J., Schulz-Schaeffer W.J., Wrede A., Koppe K., Onisko B., Requena J.R., Govindarajan N. (2014). Loss of prion protein leads to age-dependent behavioral abnormalities and changes in cytoskeletal protein expression. Mol. Neurobiol..

[75] Tuzi N.L., Clarke A.R., Bradford B., Aitchison L., Thomson V., Manson J.C. (2004). Cre-loxP mediated control of PrP to study transmissible spongiform encephalopathy diseases. Genesis.

[76] Llorens F., Ferrer I., Del Rio J.A. (2014). Gene Expression Resulting from PrP Ablation and PrP Overexpression in Murine and Cellular Models. Mol. Neurobiol..

[77] Simon D., Herva M.E., Benitez M.J., Garrido J.J., Rojo A.I., Cuadrado A., Torres J.M., Wandosell F. (2014). Dysfunction of the PI3K-Akt-GSK-3 pathway is a common feature in cell culture and in vivo models of prion disease. Neuropathol. Appl. Neurobiol..

[78] Gavin R., Braun N., Nicolas O., Parra B., Urena J.M., Mingorance A., Soriano E., Torres J.M., Aguzzi A., del Rio J.A. (2005). PrP(106-126) activates neuronal intracellular kinases and Egr1 synthesis through activation of NADPH-oxidase independently of PrP^c^. FEBS Lett..

[79] Perez M., Rojo A.I., Wandosell F., Diaz-Nido J., Avila J. (2003). Prion peptide induces neuronal cell death through a pathway involving glycogen synthase kinase 3. Biochem. J..

[80] Bautista M.J., Gutierrez J., Salguero F.J., Fernandez de Marco M.M., Romero-Trevejo J.L., Gomez-Villamandos J.C. (2006). BSE infection in bovine PrP transgenic mice leads to hyperphosphorylation of tau-protein. Vet. Microbiol..

[81] Asuni A.A., Perry V.H., O’Connor V. (2010). Change in tau phosphorylation associated with neurodegeneration in the ME7 model of prion disease. Biochem. Soc. Trans..

[82] Kapaki E., Kilidireas K., Paraskevas G.P., Michalopoulou M., Patsouris E. (2001). Highly increased CSF tau protein and decreased beta-amyloid (1-42) in sporadic CJD: A discrimination from Alzheimer’s disease?. J. Neurol. Neurosurg. Psychiatry.

[83] Sarac H., Hajnsek S., Basic S., Henigsberg N., Rados M., Simic G. (2008). Magnetic resonance spectroscopy and measurement of tau epitopes of autopsy proven sporadic Creutzfeldt-Jakob disease in a patient with non-specific initial EEG, MRI and negative 14-3-3 immunoblot. Coll. Antropol..

[84] Ishizawa K., Komori T., Shimazu T., Yamamoto T., Kitamoto T., Shimazu K., Hirose T. (2002). Hyperphosphorylated tau deposition parallels prion protein burden in a case of Gerstmann-Straussler-Scheinker syndrome P102L mutation complicated with dementia. Acta Neuropathol..

[85] Ishiguro K., Shiratsuchi A., Sato S., Omori A., Arioka M., Kobayashi S., Uchida T., Imahori K. (1993). Glycogen synthase kinase 3 beta is identical to tau protein kinase I generating several epitopes of paired helical filaments. FEBS Lett..

[86] Hernandez F., de Barreda E.G., Fuster-Matanzo A., Lucas J.J., Avila J. (2010). GSK3: A possible link between beta amyloid peptide and tau protein. Exp. Neurol..

[87] Leroy K., Yilmaz Z., Brion J.P. (2007). Increased level of active GSK-3beta in Alzheimer’s disease and accumulation in argyrophilic grains and in neurones at different stages of neurofibrillary degeneration. Neuropathol. Appl. Neurobiol..

[88] Blalock E.M., Geddes J.W., Chen K.C., Porter N.M., Markesbery W.R., Landfield P.W. (2004). Incipient Alzheimer’s disease: Microarray correlation analyses reveal major transcriptional and tumor suppressor responses. Proc. Natl. Acad. Sci. USA.

[89] Petersen R.C. (2004). Mild cognitive impairment as a diagnostic entity. J. Intern. Med..

[90] Hane F.T., Robinson M., Lee B.Y., Bai O., Leonenko Z., Albert M.S. (2017). Recent Progress in Alzheimer’s Disease Research, Part 3: Diagnosis and Treatment. J. Alzheimer’s Dis..

[91] Irwin D.J. (2016). Tauopathies as clinicopathological entities. Parkinsonism Relat. Disord..

[92] Hara M., Hirokawa K., Kamei S., Uchihara T. (2013). Isoform transition from four-repeat to three-repeat tau underlies dendrosomatic and regional progression of neurofibrillary pathology. Acta Neuropathol..

[93] Gao Q.S., Memmott J., Lafyatis R., Stamm S., Screaton G., Andreadis A. (2000). Complex regulation of tau exon 10, whose missplicing causes frontotemporal dementia. J. Neurochem..

[94] Wang J., Gao Q.S., Wang Y., Lafyatis R., Stamm S., Andreadis A. (2004). Tau exon 10, whose missplicing causes frontotemporal dementia, is regulated by an intricate interplay of cis elements and trans factors. J. Neurochem..

[95] Idda M.L., Munk R., Abdelmohsen K., Gorospe M. (2018). Noncoding RNAs in Alzheimer’s disease. Wiley Interdiscip. Rev. RNA.

[96] Smith P.Y., Delay C., Girard J., Papon M.A., Planel E., Sergeant N., Buee L., Hebert S.S. (2011). MicroRNA-132 loss is associated with tau exon 10 inclusion in progressive supranuclear palsy. Hum. Mol. Genet..

[97] Braak H., Braak E., Bohl J., Bratzke H. (1998). Evolution of Alzheimer’s disease related cortical lesions. J. Neural Transm. Suppl..

[98] Braak H., Braak E. (1996). Evolution of the neuropathology of Alzheimer’s disease. Acta Neurol Scand. Suppl..

[99] Bueler H., Fischer M., Lang Y., Bluethmann H., Lipp H.P., DeArmond S.J., Prusiner S.B., Aguet M., Weissmann C. (1992). Normal development and behaviour of mice lacking the neuronal cell-surface PrP protein. Nature.

[100] Pratt T., Sharp L., Nichols J., Price D.J., Mason J.O. (2000). Embryonic stem cells and transgenic mice ubiquitously expressing a tau-tagged green fluorescent protein. Dev. Biol..

[101] Yoshiyama Y., Higuchi M., Zhang B., Huang S.M., Iwata N., Saido T.C., Maeda J., Suhara T., Trojanowski J.Q., Lee V.M. (2007). Synapse loss and microglial activation precede tangles in a P301S tauopathy mouse model. Neuron.

[102] Taylor A.M., Blurton-Jones M., Rhee S.W., Cribbs D.H., Cotman C.W., Jeon N.L. (2005). A microfluidic culture platform for CNS axonal injury, regeneration and transport. Nat. Methods.

[103] Laemmli U.K. (1970). Cleavage of structural proteins during the assembly of the head of bacteriophage T4. Nature.

[104] Bribian A., Fontana X., Llorens F., Gavin R., Reina M., Garcia-Verdugo J.M., Torres J.M., de Castro F., del Rio J.A. (2012). Role of the cellular prion protein in oligodendrocyte precursor cell proliferation and differentiation in the developing and adult mouse CNS. PLoS ONE.

[105] Livak K.J., Schmittgen T.D. (2001). Analysis of relative gene expression data using real-time quantitative PCR and the 2(-Delta Delta C(T)) Method. Methods.

[106] Carulla P., Bribian A., Rangel A., Gavin R., Ferrer I., Caelles C., Del Rio J.A., Llorens F. (2011). Neuroprotective role of PrP^C^ against kainate-induced epileptic seizures and cell death depends on the modulation of JNK3 activation by GluR6/7-PSD-95 binding. Mol. Biol. Cell.

