# Supplementary material for: Tau Exon 10 Inclusion by PrPC through Downregulating GSK3β Activity
Source: Int J Mol Sci. 2021 May 20;22(10):5370. doi: 10.3390/ijms22105370 (PMC8161268; doi:10.3390/ijms22105370)
Supplement: Supplementary file 1 [file ijms-22-05370-s001.zip › ijms-1214594-supplementary.pdf]

Supplemental figures, Lidón et al. 2021

This file includes Figures S1-S3 that correspond to the main manuscript text.

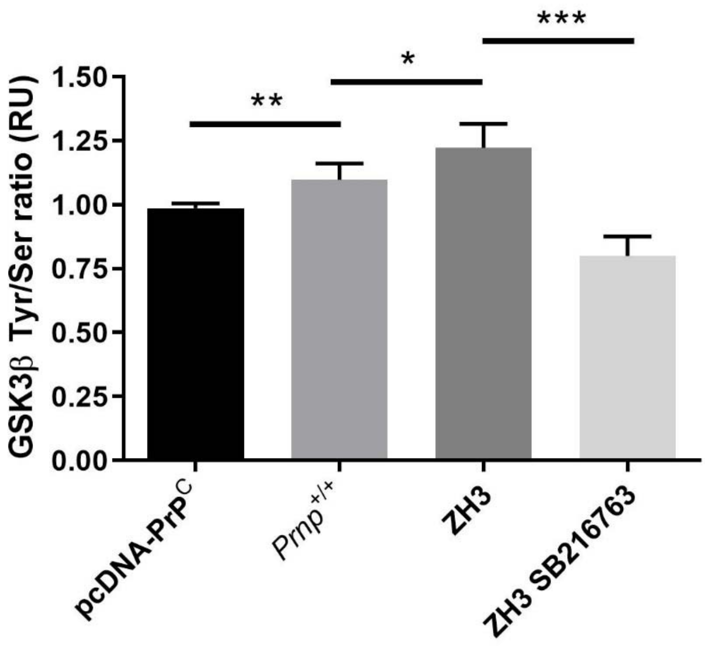

Figure S1. Changes in GSK3β activity after PrP^C overexpression in *Prnp*$^{+/+}$ neural primary cultures or using SB-216763 inhibitor in ZH3 mice cultured neurons. Bars represent GSK3β activity obtained from the ratio between phosphorylated GSK3β-$Tyr^{279/216}$ and Ser^9.

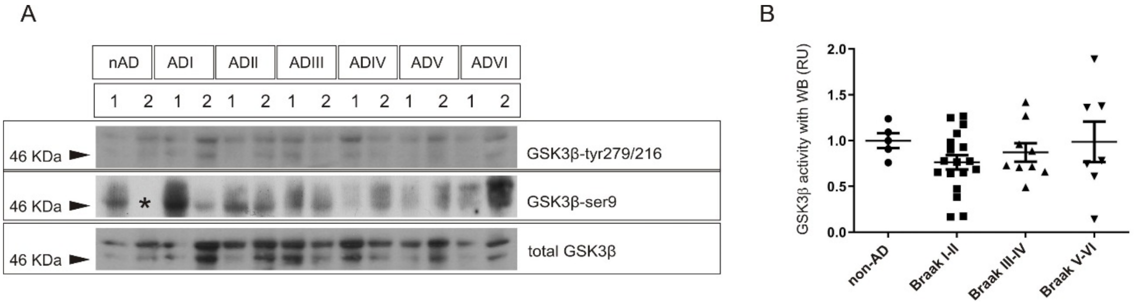

Figure S2. GSK3β activity analyzed with western blot of hippocampal necropsies from AD patients compared to healthy cases. **A.** Representative western blot analysis using anti-phospho-tyr$^{279/216}$ GSK3 antibody (monoclonal 5G-2F) in parallel with anti-phospho-ser^{9} GSK3 antibody (monoclonal 2D3) in each case. Membranes were re-probed with antibody against total GSK3 (monoclonal 4G-1E) for protein standardization. Braak and Braak stage progression is indicated (I–VI AD). Asterisk indicates an example of one sample excluded from statistical analysis for technical problems in detection. **B.** Plots illustrating the densitometric quantification of the ratio between phospho-tyr$^{279/216}$ and phospho-ser^{9} epitopes in cases shown in Table 1 corresponding to the kinase activity and grouped as Non-AD, Initial (Braak I-II), Intermediate (Braak III-IV), or Late (Braak V-VI) AD. Each dot corresponds to one sample and the mean ± SEM for each group is also displayed.

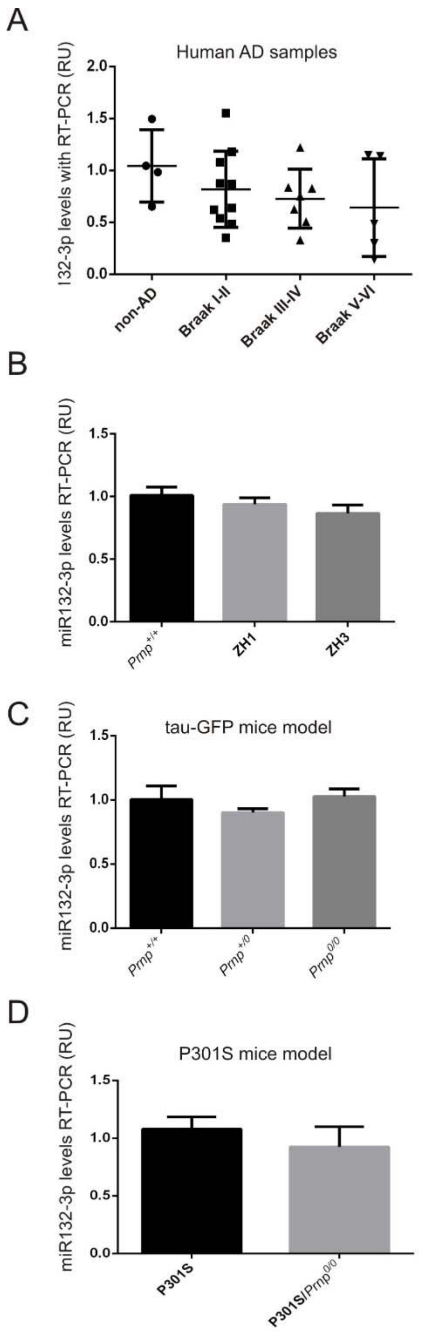

Figure S3. miR132-3p expression in human AD and mouse samples. **A.** Plots illustrating the miR132-3p expression after RT-PCR analysis in human cases shown in Table 1 and grouped as Non-AD, Initial (Braak I-II), Intermediate (Braak III-IV), or Late (Braak V-VI) AD. **B.** Histograms showing the analysis performed in WT and *Prnp*$^{0/0}$ mice ZH1 or ZH3 at the age of 3 months. **C.** Histograms showing the analysis performed in tau-GFP mouse model according to the dosage of PrP^{C} expression at the age of 3 months. **D.** Histograms showing the analysis performed in P301S and P301S-*Prnp*$^{0/0}$ mice at the age of 3 months.
